# Mammal-Skin-Inspired Adaptive Nanocomposites Cooling Membrane for Passive Battery Thermal Management

**DOI:** 10.1021/acsnano.5c11130

**Published:** 2025-09-01

**Authors:** Zengguang Sui, Jiaxiang Ma, Wei Wu

**Affiliations:** School of Energy and Environment, 53025 City University of Hong Kong, Kowloon, Hong Kong 999077, China

**Keywords:** passive battery thermal management, hygroscopic nanocomposite, desorption cooling, spontaneous absorption, porous membrane, flame retardancy, cost effectiveness

## Abstract

Efficient and flame-retardant thermal management of lithium-ion batteries (LIBs) is drawing increasing attention. Herein, we report a mammal-skin-inspired self-adaptive hygroscopic nanocomposite cooling membrane that dissipates heat from LIBs via moisture desorption and subsequently recovers its cooling capacity through spontaneous moisture absorption from ambient air. The multifunctional cooling membrane, comprising hygroscopic salt, graphene oxide, active carbon fiber, an anticorrosion copper frame, and a porous membrane, is fabricated and systematically characterized, exhibiting both outstanding cooling performance and excellent flame retardancy. Proof-of-concept experiments demonstrate that the self-adaptive cooling membrane is able to achieve an average cooling power of 802.5 W m^–2^ with a temperature reduction of 34.3 °C at a heat flux of 2.7 kW m^–2^, indicating a substantial improvement over existing passive cooling strategies with a low cost. When applied to a real 3.7 V/12 Ah LIB at a cyclic discharging-charging rate of 4C, this strategy extends the tested LIB lifetime from 118 to 233 cycles, enabling an additional total capacity of 1445.9 Ah. Long-term cycling tests at 3C reveal that the LiCl/GO@ACF membrane still maintains desirable thermal management performance after 1000 h, without risks of leakage or corrosion. Meanwhile, this cooling strategy shows superior flame retardancy and thermal stability, demonstrating the ability to inhibit thermal runaway. The flame-retardant cooling membrane developed in this study shows strong potential for enabling high-efficiency and cost-effective passive battery thermal management.

## Graphical abstract

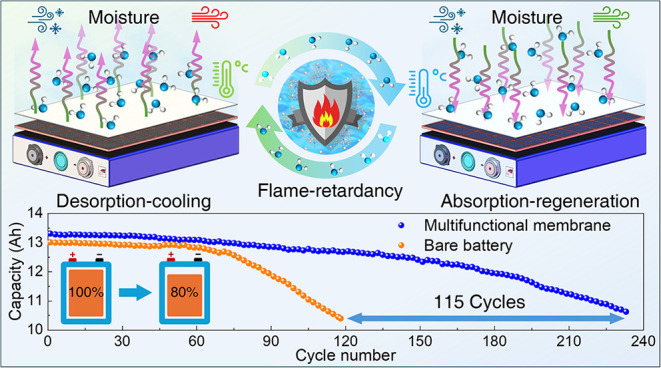

## Introduction

High-capacity rechargeable lithium-ion batteries (LIBs), as a state-of-the-art electrochemical energy storage technology, play a critical role in the ongoing transition from fossil fuels to renewable energy.^1–3^ Recently, the adoption of LIBs for consumer electronics, grid-scale energy storage, and transportation electrification continues to grow due to their high energy density, long cycle life, and low self-discharge rate.^4,5^ However, the performance of LIBs is highly sensitive to operating temperatures. Extreme operating temperatures significantly degrade LIB performance, particularly the rapid temperature rises during high-rate charging/discharging processes, which hinders widespread application. High temperatures accelerate LIB degradation, reducing reliability and lifespan, and consequently increasing replacement costs. Even worse, elevated temperatures increase the risk of thermal runaway, potentially triggering uncontrolled chain reactions that may cause fires or explosions in the absence of adequate thermal protection. Therefore, designing an efficient and reliable battery thermal management system (BTMS) is essential for maintaining safe operating temperatures.

Based on energy consumption, BTMSs are generally categorized into active and passive cooling systems. Active cooling strategies (e.g., forced air or liquid cooling) provide effective thermal regulation while suffering from additional energy consumption, bulk volume, and complex auxiliary components.^6–8^ Conversely, passive BTMSs have gained attention due to their zero energy consumption, high compactness, and low maintenance costs.^6,9–11^ Solid–liquid phase change materials (PCMs), such as paraffin waxes, can absorb substantial heat by utilizing their endothermic enthalpies during melting and are widely implemented in passive BTMSs. Research on solid–liquid PCMs mainly focused on enhancing thermal conductivity.^12^ Common approaches involve incorporating pure PCMs with highly conductive frameworks (e.g., graphite and metallic heat sinks or foams) or additives (e.g., carbon nanotubes, graphite nanoparticles).^13,14^ Despite these enhancements, large-scale PCM deployment remains limited by their low phase change enthalpies (typically <250 J g^–1^). Consequently, PCMs (∼150–250 J g^–1^) could occupy up to 50% of battery pack volume to provide latent heat capacity equivalent to the heat generated from 300 Wh kg^–1^ batteries.^15^

In nature, mammals regulate body temperature efficiently through sweating, mediated by cutaneous sweat glands. When body temperature rises, sweat glands secrete moisture onto the skin, and the subsequent evaporation absorbs substantial latent heat, thereby cooling the organism. This biological mechanism leverages the high latent heat of the liquid–vapor phase change of water (approximately 2260 J g^–1^) to achieve rapid heat dissipation. Inspired by this natural thermoregulatory strategy, water evaporation from hygroscopic materials has emerged as a promising approach for achieving high-efficiency and ultracompact BTMSs. These strategies utilize the humidity difference between the hygroscopic material surface and the surrounding air to drive vapor flow, transferring heat to the environment. Hydrogels and metal–organic frameworks (MOFs) are commonly employed as hygroscopic materials due to their high water uptake capacity and fast sorption kinetics.^16^ However, these hygroscopic materials are typically exposed to the environment without adequate encapsulation in practical applications, increasing the risks of leakage and environmental contamination during long-term operation. For hydrogel-based cooling strategies, inevitable deformation during water evaporation poses significant challenges to reliability and stability.^17–20^ Meanwhile, the flammability of the hydrogel materials also increases the risk of thermal runaway propagation once the water has fully evaporated. Although MOFs are favored for their fast sorption kinetics, large-scale application is hindered by their extremely high cost (>$100,000 kg^–1^), poor adhesion, and complex synthesis processes.^21,22^ Besides MOF-based and hydrogel-based cooling strategies, hygroscopic salt-loaded composites, which confine hygroscopic salts (i.e., calcium chloride (CaCl_2_) and lithium chloride (LiCl)) within highly hydrophilic porous matrices, are widely used for thermal management due to their low cost, high stability, facile synthesis, and high sorption capacity.^23–25^ However, these hygroscopic salt-loaded composites may absorb excessive water vapor from ambient air under high-humidity conditions, leading to solution leakage and metal corrosion.^23,26^ Furthermore, the intrinsically low thermal conductivity of these hygroscopic materials remains a fundamental limitation.^27^

Herein, inspired by the thermoregulatory mechanism of mammals, we design and fabricate a self-adaptive hygroscopic cooling membrane with fire insulation, aimed at enabling low-cost and high-efficiency passive battery thermal management. Specifically, we encapsulate a LiCl/graphene oxide (GO)-loaded active carbon fiber (LiCl/GO@ACF) composite within a breathable membrane for application in BTMSs. The uniform groove texture on the surface of each ACF fiber, combined with the strong hygroscopicity of LiCl, enables LiCl@ACF with outstanding water uptake capacity and rapid sorption kinetics. Doping GO facilitates the formation of a more efficient heat transfer network, thereby improving the heat dissipation capacity of the cooling strategy. A waterproof yet vapor-permeable porous membrane is used to encapsulate the LiCl/GO@ACF, preventing failure from solution leakage and dust pollution. As demonstrated in proof-of-concept experiments, the proposed cooling strategy achieves an average cooling power of 802.5 W m^–2^ with 34.3 °C temperature reduction, surpassing state-of-the-art passive cooling strategies that rely on water desorption from hygroscopic materials, while maintaining low cost. When applied to 3.7 V/12 Ah nickel–cobalt-manganese LIBs at a high discharging-charging rate of 4C (where 1C represents the battery fully discharging/charging within 1 h), our cooling strategy extends the battery lifetime (defined as the number of charging–discharging cycles during which LIBs maintain at least 80% of their nominal capacity) from 118 to 233 cycles, delivering an additional capacity of 1445.9 Ah without signs of corrosion or leakage. In addition to efficient heat dissipation, the LiCl/GO@ACF membrane exhibits excellent fire resistance, which is a crucial safety feature for LIB applications. The functional integration of these materials, combined with the purposeful design of the cooling membrane, addresses key thermal management challenges of LIBs and provides a new pathway for developing efficient, ultracompact, flame-retardant, and cost-effective passive thermal management systems.

## Results and Discussion

### Working Principle of the Proposed Cooling Membrane

Mammals regulate body temperature by releasing water through sweat glands distributed across their skin (Figure 1A). Inspired by this process, we design and fabricate a self-adaptive hygroscopic cooling membrane to prevent batteries from overheating and thermal runaway propagation. This cooling membrane attaches directly to the surface of LIBs for thermal management (Figure 1B). A highly thermally conductive graphene film serves as an anticorrosion interface while reducing the contact thermal resistance between the LiCl/GO@ACF and the LIB. A graphene-coated copper frame is employed to enhance heat and mass transfer performance and to prevent nonuniform solution distribution in the LiCl/GO@ACF caused by gravity (see Figure S1 for details). The working principle of the proposed cooling strategy is illustrated in Figure 1C. The strategy relies on water evaporation from the LiCl/GO@ACF membrane to prevent LIBs from overheating at high temperatures (i.e., desorption cooling). At lower temperatures, the cooling membrane spontaneously absorbs ambient moisture to regenerate its cooling capacity (i.e., absorption regeneration) via the strong hygroscopic properties of LiCl. Figure S2 schematically illustrates the desorption-absorption cycle. During the desorption-cooling process (a-b), the heat generated by LIBs is absorbed by the LiCl/GO@ACF to drive water desorption (i.e., the vapor partial pressure of the LiCl solution (*p*_s_) is higher than the ambient vapor partial pressure (*p*_v_)). Subsequently, the LiCl/GO@ACF undergoes a brief desorption process as *p*_s_ remains higher than *p*_v_ (b-c), and then begins to absorb water vapor from the surrounding air once *p*_s_ < *p*_v_ (c-a). Thus, the LiCl/GO@ACF can autonomously recover water content, enabling passive cyclic operation. Besides, a porous poly­(tetrafluoroethylene) (PTFE) membrane (see detailed parameters in Table S1) is used to encapsulate the LiCl/GO@ACF, preventing solution leakage and isolating environmental dust while allowing only water vapor to pass through, ensuring stable and efficient thermal management.

**Figure 1:**
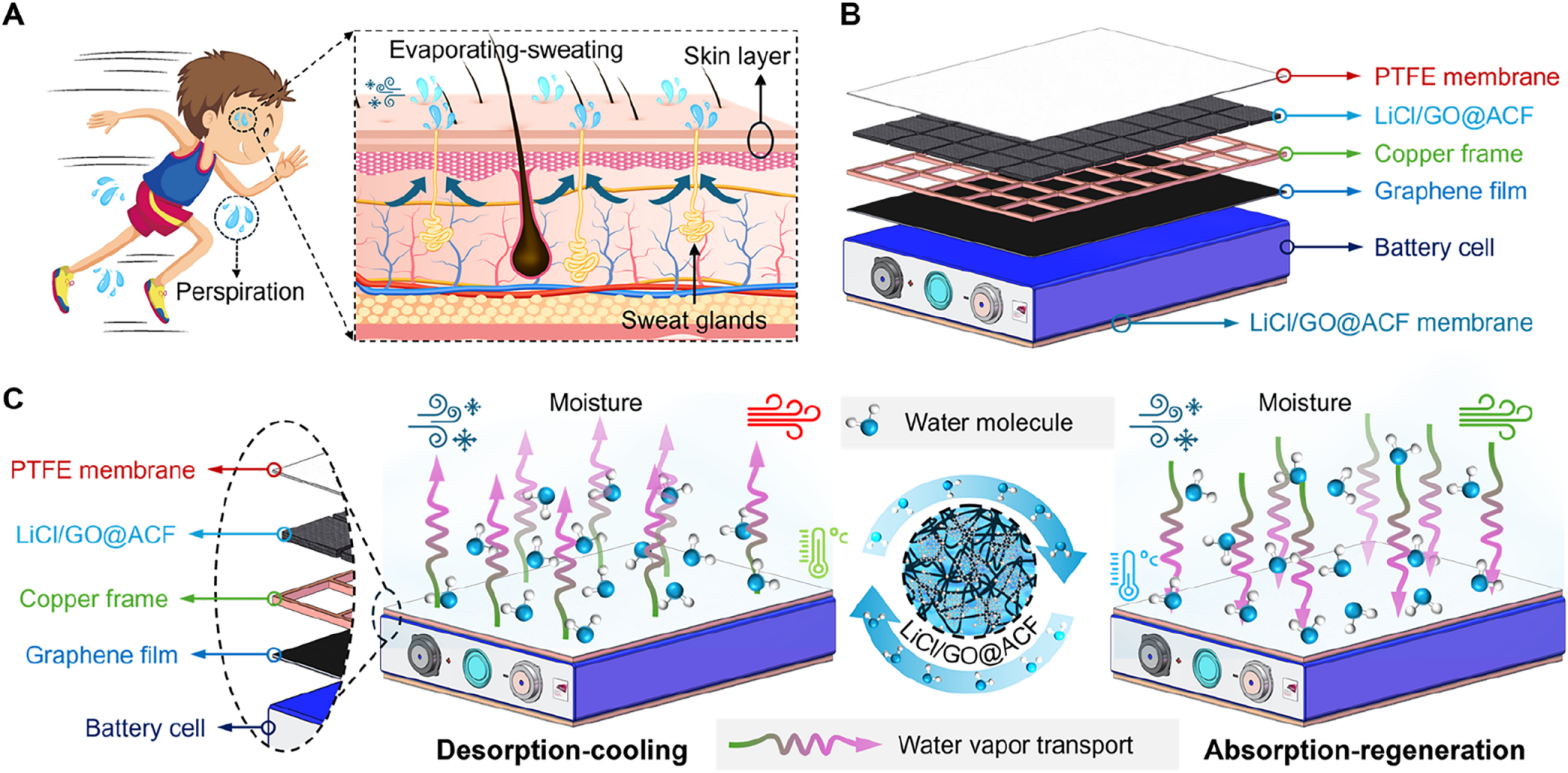
Working principle of the cooling strategy. (A) Schematic diagram showing the body heat dissipation mechanism. Mammals regulate body temperature by releasing water through sweat glands distributed across their skin. (B) Schematic illustration of the proposed cooling strategy applied to LIBs (see Figure S1 for details). (C) Desorption and absorption cycles of the proposed strategy. At high temperatures, the water evaporation from the LiCl/GO@ACF membrane provides desorption cooling effects (desorption-cooling process), while the LiCl/GO@ACF membrane absorbs the moisture from the surrounding air to recover its water contents at low temperatures (absorption-regeneration process).

To explore the heat dissipation mechanism of the LiCl/GO@ACF membrane more thoroughly, a simplified energy balance model is developed. According to the principle of energy conservation, the energy balance equation can be expressed as

1 $$Q_{\mathrm{gen}}=A\sigma \varepsilon \left(T_{s}^{4}-T_{\mathrm{amb}}^{4}\right)+Ah_{\mathrm{nat}}\left(T_{s}-T_{\mathrm{amb}}\right)+ṁh_{\mathrm{des}}+\sum m_{i}C_{i}\frac{dT_{i}}{dt}$$

where *A* is the surface area; σ is the Stefan–Boltzmann constant; ε is the emittance; *T*_s_ is the surface temperature; *T*_amb_ is the ambient temperature; ṁ is the desorption rate; *h*_des_ is the desorption enthalpy; *m*_*i*_, *T*_*i*_, and *C*_*i*_ are the mass, temperature, and specific heat capacity of each part of the cooling strategy, respectively; *Q*_gen_ represents the heat generated from LIBs including the electrochemical reaction heat and the heat from the internal resistance of LIBs,^8^ which can be given as

2 $$Q_{\mathrm{gen}}=I^{2}R-IT_{\mathrm{cell}}\frac{dU_{\mathrm{oc}}}{dT_{\mathrm{cell}}}$$

where *I*, *R*, *T*_cell_, and *U*_oc_ denote the current, internal resistance, battery cell temperature, and open-circuit voltage, respectively.

Compared to traditional passive heat dissipation methods for LIBs, such as surface radiation and convection, the proposed strategy incorporates an additional cooling mechanism via water evaporation from LiCl/GO@ACF (i.e., the third term on the right side of eq 1). The high evaporation enthalpy of the LiCl/GO@ACF (>2000 J g^–1^), which is ∼10 times that of conventional solid–liquid PCMs (commonly <250 J g^–1^), endows the proposed cooling strategy with exceptional thermal regulation potential for practical applications characterized by intermittent and fluctuating heat generation.

### Preparation and Characterization of LiCl/GO@ACF Membrane

The fabrication process of the LiCl/GO@ACF membrane is depicted in Figure 2A (additional details provided in Figure S1). A GO solution with a concentration of 5 mg mL^–1^ was mixed with a prepared 25% LiCl solution to form a homogeneous LiCl/GO mixture via magnetic stirring for 12 h. Subsequently, ACF felt was immersed in the LiCl/GO solution for 12 h to produce the LiCl/GO@ACF composite. To systematically evaluate the effect of GO content on cooling performance, LiCl/GO@ACF samples with GO contents varying from 0.5%–5.0% were prepared. The LiCl/GO@ACF composites with varying GO contents were thoroughly dried and subsequently placed on a high-precision electronic balance in an environmental chamber (RH = 60% and *T*_amb_ = 25 °C) to measure their water uptake capacity (see Note S1 for details). Results indicate that the water uptake capacity of the LiCl/GO@ACF decreases sharply when the GO content exceeds 2.5% (Figure 2B). Furthermore, thermal conductivity measurements (Figure 2B) reveal that increasing GO content enhances the thermal conductivity of the LiCl/GO@ACF. This improvement is attributed to the efficient heat transfer network formed by the doped GO sheets. Considering both water uptake capacity and thermal conductivity, the LiCl/GO@ACF composite with 2.5% GO content was ultimately selected. The mechanical performance of the selected LiCl/GO@ACF demonstrates that it can be used for LIBs with different 3D geometries (e.g., cylindrical, rectangular, and pouch cells) due to its outstanding soft and integral features during deformation (Figure S3).

**Figure 2:**
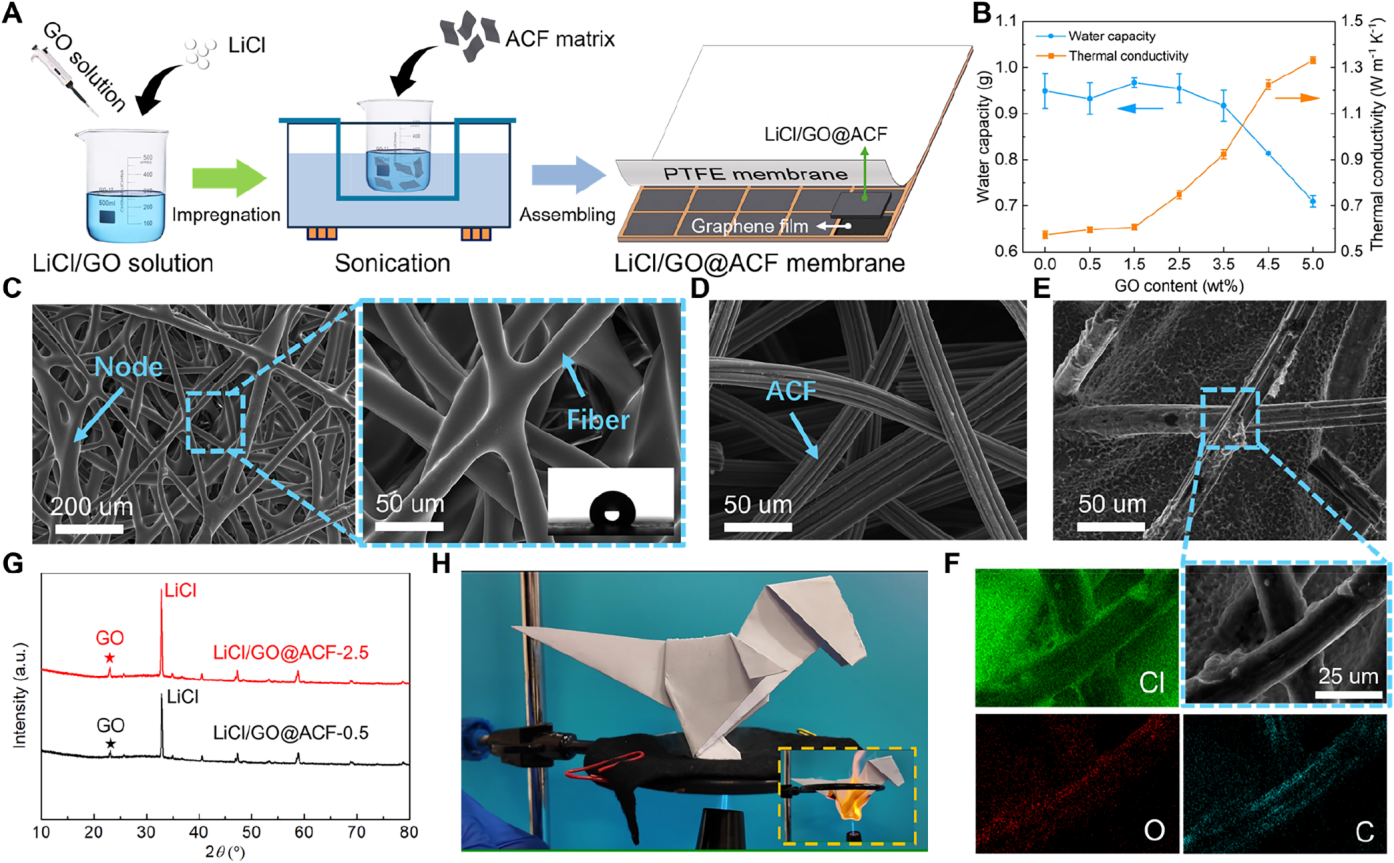
Design and characterization of the LiCl/GO@ACF membrane. (A) Schematic diagram showing the fabrication process of the LiCl/GO@ACF membrane. (B) Water uptake capacities and thermal conductivities of the LiCl/GO@ACF with different GO contents (see Note S1 for water vapor absorption experiments). Error bars represent the standard deviation (*n* = 3). (C) SEM images of the PTFE membrane, and the inset shows the contact angle of LiCl solution with the PTFE membrane (more details in Figures S4 and S5). (D) SEM image of the original ACF matrix, showing the 3D interconnected pores formed by fibers (more details in Figure S6A). (E) SEM image of the LiCl/GO@ACF to show the distribution of LiCl and GO (more details in Figure S6B). (F) EDS elemental mapping of the LiCl/GO@ACF to demonstrate the uniform distribution of LiCl and GO. (G) XRD patterns of the LiCl/GO@ACF with GO content of 0.5% and 2.5%, demonstrating LiCl and GO are successfully incorporated into the ACF matrix, which can be further verified by the XPS results (see Figure S7 for XPS measurements). (H) Photograph showing the fire resistance property of the LiCl/GO@ACF. The inset shows that the paper origami dinosaur is flammable (see details in Videos S1 and S2).

The selected LiCl/GO@ACF was encapsulated in an anticorrosion copper frame using a porous PTFE membrane with a pore diameter of 1 μm and a graphene film. The PTFE membrane, composed of fibers and nodes (Figure 2C, more details in Figure S4), shows strong hydrophobicity (Figure S5). Meanwhile, the scanning electron microscopy (SEM) image of the original ACF reveals a uniform groove texture on the surface of each fiber (Figure 2D), which enhances the attachment of LiCl/GO, thereby improving the heat and mass transfer performance.^28^ The morphology of LiCl/GO@ACF with 2.5% GO content (LiCl/GO@ACF-2.5) was characterized in Figure 2E,F. The SEM image (Figure 2E) and energy dispersive X-ray spectroscopy (EDS) element mapping (Figure 2F) demonstrate the uniform distribution of LiCl and GO on the fiber surface. This uniform distribution increases the sorption area and improves thermal conductivity, thereby enhancing the cyclic working capacity (more details in Figure S6). The X-ray diffraction (XRD) analysis of LiCl/GO@ACF-0.5 and LiCl/GO@ACF-2.5 (Figure 2G) reveals distinct peaks corresponding to LiCl and GO, confirming their successful incorporation into the ACF matrix, which is further verified by the X-ray photoelectron spectra (XPS) of the pure ACF and dried LiCl/GO@ACF (Figure S7). Additionally, the XPS analysis indicates a substantial enrichment of polar oxygen-containing groups (C–O and C=O) on the LiCl/GO@ACF compared to the pure ACF. These sites can facilitate hygroscopicity via hydrogen bonding and dipole–dipole interactions. After ultrasonication, the oxygen content decreases substantially but remains above that of the pure ACF, indicating partial retention of GO. In contrast, Li and Cl signals decrease to levels indistinguishable from the pure ACF background, demonstrating that LiCl is predominantly retained via physical adsorption or confinement. To measure the stability of the LiCl/GO@ACF, a long-term cycling aging experiment was conducted. Figure S8 demonstrates that the LiCl/GO@ACF could maintain a desired water uptake capacity after 100 cycles.

To evaluate the flame-retardant ability of the LiCl/GO@ACF, we conducted burning experiments. Paper placed on dried LiCl/GO@ACF remains intact without any signs of combustion (Figure 2H and Video S1), while the unprotected paper ignites and burns immediately (the inset of Figure 2H and Video S2). To further evaluate the flame-retardant property of the LiCl/GO@ACF, we conducted the limiting oxygen index (LOI) test based on the test standard ISO 4589. Table S2 indicates that LiCl/GO@ACF remains unburned at an oxygen concentration of 90%, demonstrating its exceptional fire resistance ability. Besides, the combustion behavior of the LiCl/GO@ACF was further analyzed using a cone calorimeter. Interestingly, LiCl/GO further enhances the flame retardancy of the ACF. The peak heat release rate (PHRR) of the LiCl/GO@ACF declines to 14.64 kW m^–2^ at 153 s, while its total heat release (THR) decreases to 8.31 MJ m^–2^ at 1800 s (see Figure S9 and Table S3). The extremely low PHRR and THR demonstrate the excellent flame-retardant properties of LiCl/GO@ACF. The unique incombustible properties of LiCl/GO@ACF can be attributed to the intrinsic high thermal stability of LiCl/GO and carbon fiber.^29–33^ Meanwhile, ignition and flame times listed in Table S3 further confirm the results obtained from the LOI, i.e., the LiCl/GO@ACF does not ignite under the tested conditions. These findings suggest that LiCl/GO@ACF can effectively prevent fire propagation caused by thermal runaway, providing critical time for active control measures.

### Proof-of-Concept Experiments of LiCl/GO@ACF Membrane

To evaluate the feasibility of the cooling membrane, we conducted proof-of-concept experiments. We used a polyimide (PI) heater to provide stable heat flux for simulating the heat generation of LIBs during discharging/charging. The heat flux was regulated by a direct-current power supply. The prepared samples were measured using an anticorrosion aluminum heat sink (Figure S10), and a high-precision electronic balance recorded the mass changes of the LiCl/GO@ACF during the desorption-absorption process. All experiments were conducted in an environment-controlled chamber (Figure 3A, see Note S1 for details). To investigate the effect of GO content on the sorption capacity, the dynamic sorption performance of LiCl/GO@ACF with varying GO contents was measured for 20 h (Figure 3B, see Note S1 for details). When the GO content exceeds 2.5%, the doped GO significantly affects the dynamic sorption process and water content of LiCl/GO@ACF. This effect can be attributed to a reduced effective contact area between LiCl crystals and the surrounding air due to the GO-covered ACF fiber surface and the agglomeration of GO sheets.

**Figure 3:**
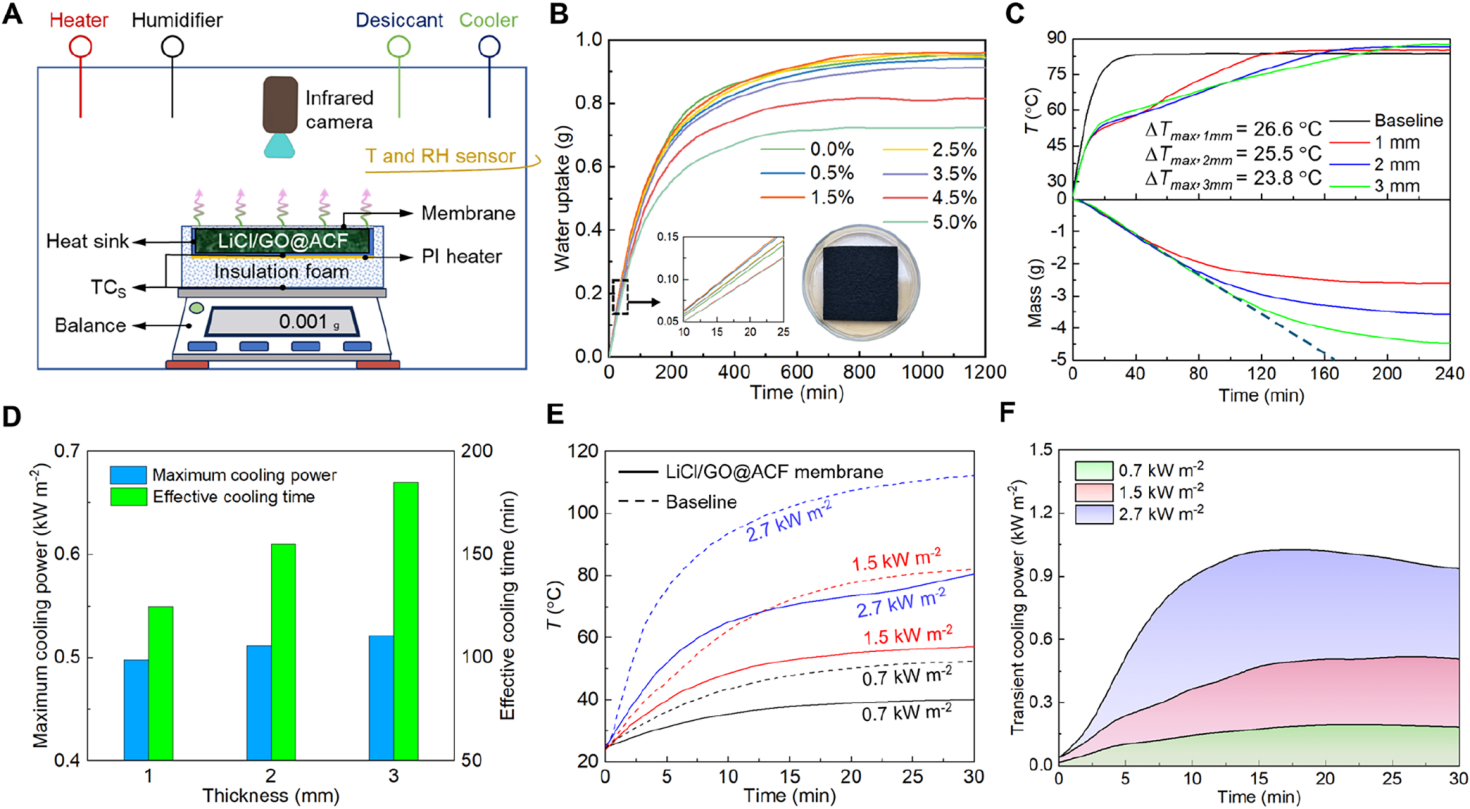
Cooling performance of LiCl/GO@ACF membrane (RH = 60% and *T*_amb_ = 25 °C). (A) Schematic of the test environment-controlled chamber. (B) Water uptake capacity of the LiCl/GO@ACF with different GO contents (see Note S2 for details). (C) Temperature evolution of the heater and mass evolution with the LiCl/GO@ACF membrane with different thicknesses at a heat flux of 1.5 kW m^–2^. (D) Comparison of the cooling performance in terms of the maximum latent cooling power (i.e., the cooling power provided by water evaporation) and the effective cooling time. (E) Temperature evolution of the heater at varying heat fluxes using the LiCl/GO@ACF membrane with a thickness of 2 mm. (F) Comparison of the transient cooling power at varying heat fluxes. The transient cooling power was obtained by deriving from the corresponding mass curves.

Prior to the experiments, the LiCl/GO@ACF encapsulated in the porous PTFE membrane (i.e., LiCl/GO@ACF membrane) was dried for 12 h and then placed in an environment-controlled chamber for 24 h (RH = 60% and *T*_amb_ = 25 °C). At a heat flux of 1.5 kW m^–2^, the mass and temperature evolutions of the samples with different thicknesses were recorded (Figure 3C). Compared to the baseline (Figure S10), the proposed cooling strategy effectively slows down the temperature rise of the heater. This is attributed to the rapid water desorption from the LiCl/GO@ACF membrane, as evidenced by the mass loss curves in Figure 3C. The temperature gradually peaks as the mass loss of the samples slows down. The maximum temperature reduction between the baseline and the LiCl/GO@ACF membrane decreases from 26.6 to 23.8 °C as the LiCl/GO@ACF membrane thickness increases from 1 mm to 3 mm. It is noteworthy that the initial mass decreases linearly with a consistent slope for all three samples at the fixed heat flux (see mass curves in Figure 3C), indicating that their mass loss rates (i.e., desorption rate) are nearly identical regardless of the LiCl/GO@ACF membrane thickness. This phenomenon is attributed to the fact that the water vapor transfer resistance of the membrane dominates the fast desorption process at the fixed heat flux.^34,35^ For comparison, we obtained the temperature evolutions of the samples with various cooling strategies, including the baseline, GO@ACF membrane, LiCl@ACF, LiCl/GO@ACF, LiCl/GO@ACF membrane, a hydrogel-based cooling strategy, and a traditional PCM (Figure S11). Compared to the LiCl@ACF cooling strategy, doping GO is expected to improve the cooling performance. Despite introducing additional thermal resistance, the temperature difference between the LiCl/GO@ACF membrane and the PCM cooling strategy (i.e., paraffin wax) can reach up to 26.1 °C, while the temperature difference between the LiCl/GO@ACF membrane and the LiCl@PAM can reach 8.7 °C.

To quantitatively discuss the effect of the LiCl/GO@ACF membrane thickness on the effective thermal management duration, an effective cooling time was defined as the time at which the sample temperature exceeds that of the baseline. As shown in Figure 3D, the effective cooling time increases from 125 to 185 min as the LiCl/GO@ACF membrane thickness increases from 1 mm to 3 mm, indicating a positive correlation between effective cooling time and LiCl/GO@ACF membrane thickness. Thinner LiCl/GO@ACF membranes exhibit lower thermal resistance, resulting in a higher temperature reduction but shorter effective cooling time. Additionally, the transient cooling power was derived from the mass curves. The maximum cooling power increases from 0.50 kW m^–2^ to 0.53 kW m^–2^ with an increase in the LiCl/GO@ACF membrane thickness from 1 mm to 3 mm. These values exceed one-third of the total heat flux (1.5 kW m^–2^), highlighting the superior cooling effect of the proposed strategy.

Figure 3D clearly shows that increasing the LiCl/GO@ACF membrane thickness contributes to extending the effective cooling time. However, thicker LiCl/GO@ACF membranes introduce higher thermal resistance. To balance effective cooling time and thermal resistance, we measured the cooling performance of the LiCl/GO@ACF membrane with a thickness of 2 mm under different heat fluxes (Figure 3E). Higher heat fluxes result in sharper temperature rises, corresponding to greater maximum temperature reductions. Meanwhile, the transient cooling power supplied by the water desorption in the proposed strategy is influenced by the heat flux (Figure 3F). A maximum transient cooling power of 1.03 kW m^–2^ is achieved at a heat flux of 2.7 kW m^–2^. Additionally, we defined an average cooling power (*q*) provided by the water desorption from the LiCl/GO@ACF membrane to assess the cooling performance of the proposed strategy as follows

3 $$q=\frac{\Delta m\times h_{\mathrm{des}}}{t\times A_{m}}$$

where Δ*m* is the mass loss of the LiCl/GO@ACF membrane during the desorption process; *t* is the desorption duration; *A*_*m*_ is the surface of the PTFE membrane. The desorption enthalpy of the LiCl/GO@ACF membrane, determined through differential scanning calorimetry and thermogravimetric analysis, is 2278.1 J g^–1^ (Figure S12). During the desorption-cooling process for 30 min, the average cooling power is 0.16 kW m^–2^, 0.42 kW m^–2^, and 0.90 kW m^–2^ at heat fluxes of 0.7 kW m^–2^, 1.5 kW m^–2^, and 2.7 kW m^–2^, respectively.

### Cyclic Performance of LiCl/GO@ACF Membrane

To ensure efficient cooling performance under intermittent workloads, the proposed cooling strategy should possess not only a fast desorption rate but also an excellent moisture absorption rate to quickly recover its cooling capacity. We measured the dynamic water uptake performance of the LiCl/GO@ACF membrane under varying RH conditions at a temperature of 25 °C (see Note S1 for details). As shown in Figure 4A, higher ambient humidity leads to a faster absorption rate, enabling a higher water content. Notably, the cooling strategy still maintains excellent moisture absorption capacity even at lower RH conditions (RH = 25%), which is attributed to the increased absorption area of the highly porous ACF matrix. These results suggest that the proposed strategy can be effectively applied in diverse climate conditions due to its superior water vapor absorption capacity.

**Figure 4:**
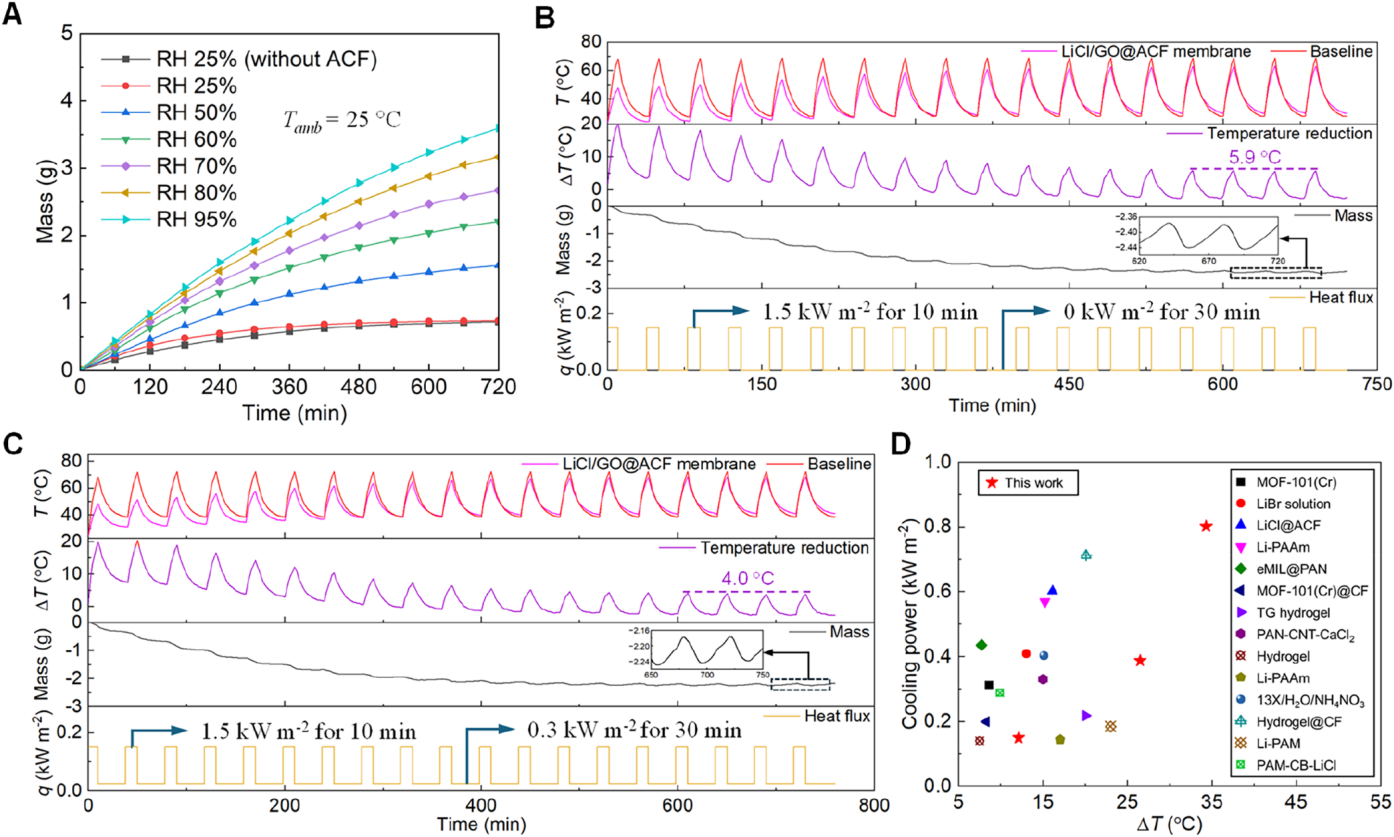
Cooling performance with varying working conditions. (A) Dynamic water uptake capacity of the LiCl/GO@ACF membrane under different RH conditions (*T*_amb_ = 25 °C). (B) Cooling performance of the proposed strategy under periodic workloads by switching between 1.5 kW m^–2^ and 0 kW m^–2^ at RH = 50% and *T*_amb_ = 25 °C. (C) Cooling performance of the proposed strategy under periodic workloads at RH = 50% and *T*_amb_ = 25 °C. (D) Cooling performance comparison of the strategy and previously reported passive cooling strategies. The detailed testing conditions for different passive cooling strategies are listed in Table S4.

To further investigate the impact of absorption-regeneration time on the cooling performance, we conducted cycling experiments under intermittent workloads. Figure 4B compares the cooling performance of the proposed strategy with the baseline at a low humidity of 50% (heat flux: 1.5 kW m^–2^ for 10 min and 0 kW m^–2^ for 30 min). The cooling efficiency gradually declines during the initial cycles, with the maximum peak temperature reduction ultimately stabilizing at 5.9 °C. This decline is attributed to incomplete water regeneration in the LiCl/GO@ACF membrane. Notably, the mass continues to decrease (i.e., the LiCl/GO@ACF membrane remains in a desorption process) after the heat flux is switched to 0 kW m^–2^ during the initial cycles. This phenomenon can be explained by the pressure–temperature-concentration lines of the LiCl solution shown in Figure S2 (i.e., *p*_s_ > *p*_v_). In Figure S13, we conducted two additional cycling experiments to assess cooling performance with shorter regeneration times. Results show that the stable peak temperature reduction decreases from 5.9 to 5.3 °C as the regeneration time is reduced from 30 to 10 min. Furthermore, we measured the regeneration capacity of the cooling strategy under a low workload, as shown in Figure 4C. The heat flux was alternated between 1.5 kW m^–2^ for 10 min and 0.3 kW m^–2^ for 30 min, emulating high and low heat generation, respectively. Although the cooling performance slightly deteriorates compared to the foregoing experiment (Figure 4B) due to the decreased cyclic water uptake under lower workloads, the stable peak temperature reduction remains as high as 4.0 °C. These experiments demonstrate that the proposed strategy provides efficient cooling capacity even when the water content in the LiCl/GO@ACF membrane is incompletely recovered. Notably, no visible signs of corrosion or leakage were observed during the cycling experiments.

Additionally, standardized evaluation criteria for comparing different cooling strategies remain absent due to varying testing conditions. We conducted a comprehensive comparison to evaluate the cooling performance and cost effectiveness of various passive cooling strategies, with detailed testing conditions provided in Table S4. Considering the temperature reduction and cooling power, Figure 4D demonstrates that our proposed cooling strategy is expected to offer superior cooling performance compared to previous studies.^17–19,23,36–45^ Note S2 provides a detailed cost analysis based on the raw materials used in the LiCl/GO@ACF membrane. The raw material cost of the cooling membrane is approximately 11.53 USD m^–2^, which is 5.31% of the LIB price (3.58 USD for the tested battery). It should be mentioned that the LiCl/GO@ACF membrane fabrication process is simple and energy-efficient, unlike hydrogel and MOF cooling strategies, which require complex manufacturing processes (freeze-drying treatment and vacuum treatment). These results highlight the potential of the proposed cooling membrane for large-scale commercialization.

### Performance Demonstration of LiCl/GO@ACF Membrane on Batteries

We applied the LiCl/GO@ACF membrane to a commercial 3.7 V/12 Ah nickel–cobalt-manganese LIB to further evaluate the cooling performance of the proposed strategy (Figure S14). The LIB parameters are detailed in Table S5. Specifically, the LIB, wrapped with the LiCl/GO@ACF membrane, was tested in an environmental chamber (RH = 60% and *T*_amb_ = 25 °C). A thermocouple was mounted at the center of the LIB surface to monitor temperature changes. The detailed experimental setup is described in Note S1. As shown in Figure 5A, the LIB equipped with the LiCl/GO@ACF membrane shows a lower temperature compared to the bare LIB during 3C discharging, with a maximum temperature reduction of up to 9.8 °C. At a discharging rate of 4C, the maximum temperature reduction reaches 14.4 °C (Figure 5B). Additionally, we compared the LiCl/GO@ACF membrane with a PCM cooling strategy to demonstrate the superiority of our proposed thermal management strategy (Figure S15). In Figure 5A,B, the maximum temperature reductions are increased by up to 3.3 °C (3C) and 4.3 °C (4C) compared to the PCM cooling strategy. Meanwhile, the real-time temperature distribution of the LIB during 4C discharging was visualized using an infrared (IR) camera (Figure 5C). The water desorption from the LiCl/GO@ACF membrane rapidly dissipates the heat generated by the LIB, yielding lower temperatures in the LiCl/GO@ACF regions than in the copper frame regions.

**Figure 5:**
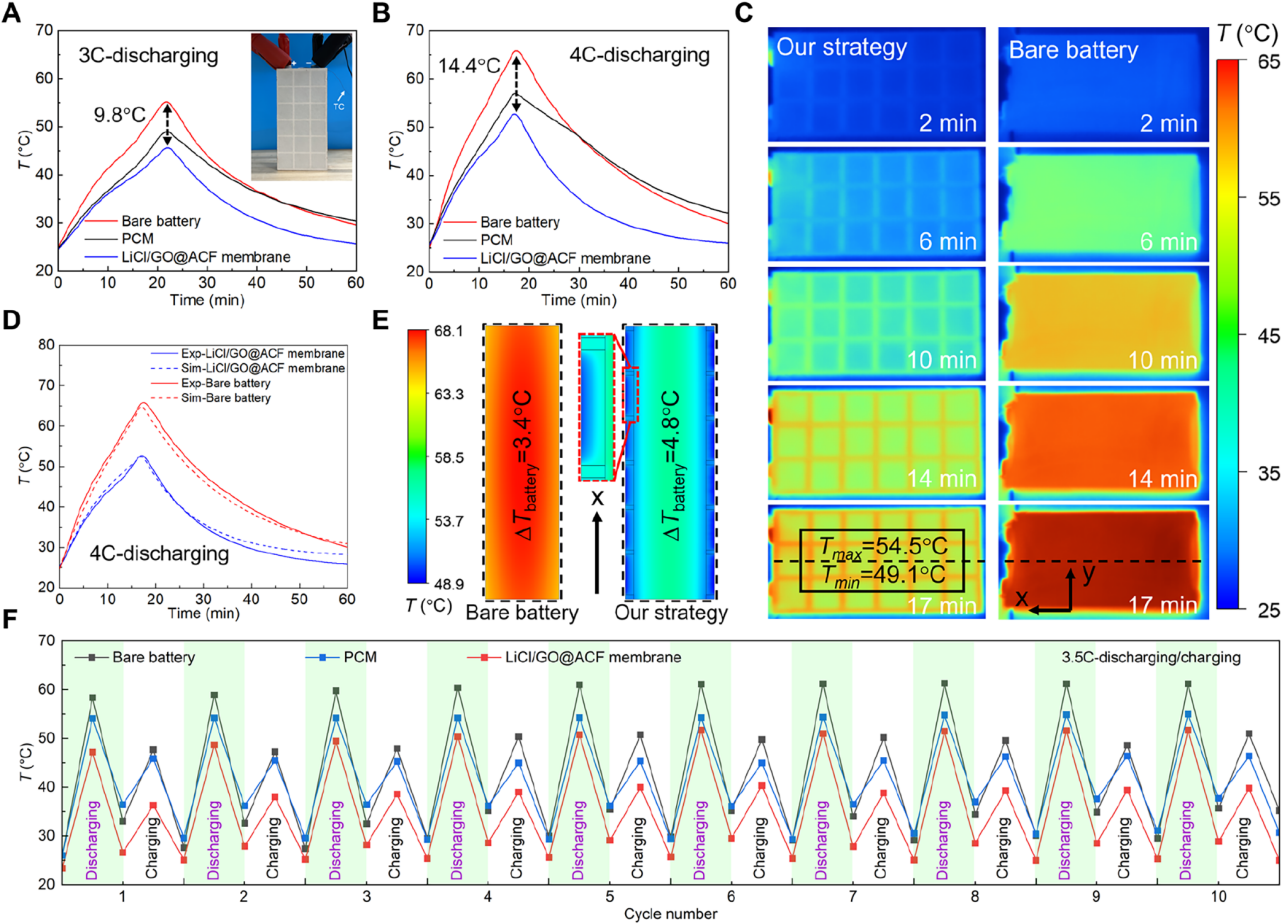
Demonstration of the LiCl/GO@ACF membrane to cool the commercial LIB. (A) Temperature evolution of the LIBs with and without the LiCl/GO@ACF membrane at a discharging rate of 3C. The inset shows the LIB with the LiCl/GO@ACF membrane (see details in Figure S14). (B) Temperature evolution of the LIBs with and without the LiCl/GO@ACF membrane at a discharging rate of 4C. (C) IR images of the LIB with and without the LiCl/GO@ACF membrane over time. (D) Comparison of experimental and simulation results for the LIB with the LiCl/GO@ACF membrane at a discharging rate of 4C. (E) Simulated temperature contours at the cross section (black dashed lines in (C)). The scale factor is 0.5 in the *x* direction to show the contours more clearly, and the detailed temperature contours over time can be found in Figure S17. (F) Temperature evolution of the LIBs with and without the LiCl/GO@ACF membrane at a discharging-charging rate of 3.5C in 10 cycles.

To analyze the internal temperature distribution of the LIB, we developed the heat and mass transfer models of the LIB wrapped with the LiCl/GO@ACF membrane (details provided in Note S3). The simulated temperature evolution agrees well with the experimental results (Figure 5D), indicating the high accuracy of the developed numerical model. Using the experimentally validated numerical model, we observe that the maximum temperature increases from 51.8 to 55.4 °C as the PTFE membrane pore diameter decreases from 1.5 to 0.45 μm (Figure S16A). Decreasing the membrane pore diameter can improve the pressure potential (*p*_s_ – *p*_v_) (Figure S16B), however, it increases the mass transfer resistance through the membrane, resulting in a decrease in the mass flux (*J*) (Figure S16C) and therefore a lower LiCl mass fraction (Figure S16D). The results obtained from Figure S16 confirm the conclusion drawn from Figure 3C (i.e., the water vapor transfer resistance caused by the porous membrane dominates desorption at given heat flux). According to the simulated cross-sectional temperature contours (Figure 5E, black dashed lines in Figure 5C, see details in Figure S17), the maximum temperature difference of the battery at the 4C discharging rate is 4.8 °C (generally <5 °C). This demonstrates desirable temperature uniformity even at high discharge rates. Notably, the optimal design configuration of the LiCl/GO@ACF membrane can be determined using the numerical model based on specific operational requirements.

To demonstrate the superiority of the proposed cooling strategy over traditional PCMs, we compare temperature evolution between LIBs equipped with the LiCl/GO@ACF membrane and a PCM cooling strategy, as depicted in Figure 5F (RH = 65% and *T*_amb_ = 25 °C). Considering the melting point of the PCM and aiming to avoid the influence of premature battery aging due to high temperatures on temperature measurements, the experiment was conducted at a discharging-charging rate of 3.5C with 10 cycles. The battery with our cooling membrane can always operate at lower peak temperatures than both bare and PCM-cooled LIBs. The maximum temperature of the LIB with the LiCl/GO@ACF membrane is only 52.1 °C, within the acceptable temperature range for the LIB.^39,46^ By maintaining the LIB at a suitable operating temperature, the cooling membrane enables deeper charging–discharging cycles, thereby extending effective LIB working time (e.g., enlarging the driving range for electric vehicles). Meanwhile, the peak temperature differences between the bare battery and the battery with the LiCl/GO@ACF membrane in each cycle are displayed in Figure S18. These temperature differences are maintained between 9.1 and 11.4 °C, further confirming the superior cooling performance of our proposed LiCl/GO@ACF membrane.

LIBs typically reach end-of-life (EoL) points when the battery capacity deteriorates to 80% of its nominal capacity.^46,47^ To assess the long-term cycling performance of the cooling strategy for practical applications, the cycling performance of the LIB with the LiCl/GO@ACF membrane was measured at a cyclic discharging-charging rate of 3C. The specific operating conditions during cyclic testing are provided in Table S6. Figure 6A presents the practical thermal management performance of the cooling membrane. The cooling membrane effectively maintains maximum temperatures below 47.5 °C, significantly lower than the bare battery (Figure S19). Benefiting from the temperature reduction, the battery with the LiCl/GO@ACF membrane retains 90.5% capacity after 462 cycles (∼1000 h) without any solution leakage, whereas the bare battery reaches its EoL point after only 196 cycles. Meanwhile, Figure 6B characterizes the mechanical strength and microstructure of the PTFE membrane after the long-term cycling (see Figure S20 for details). The PTFE membrane shows excellent mechanical properties with a breaking strength of ∼21.8 MPa and an intact microstructure, indicating outstanding stability and sufficient protection for the LiCl/GO@ACF composite in real-world applications.

**Figure 6:**
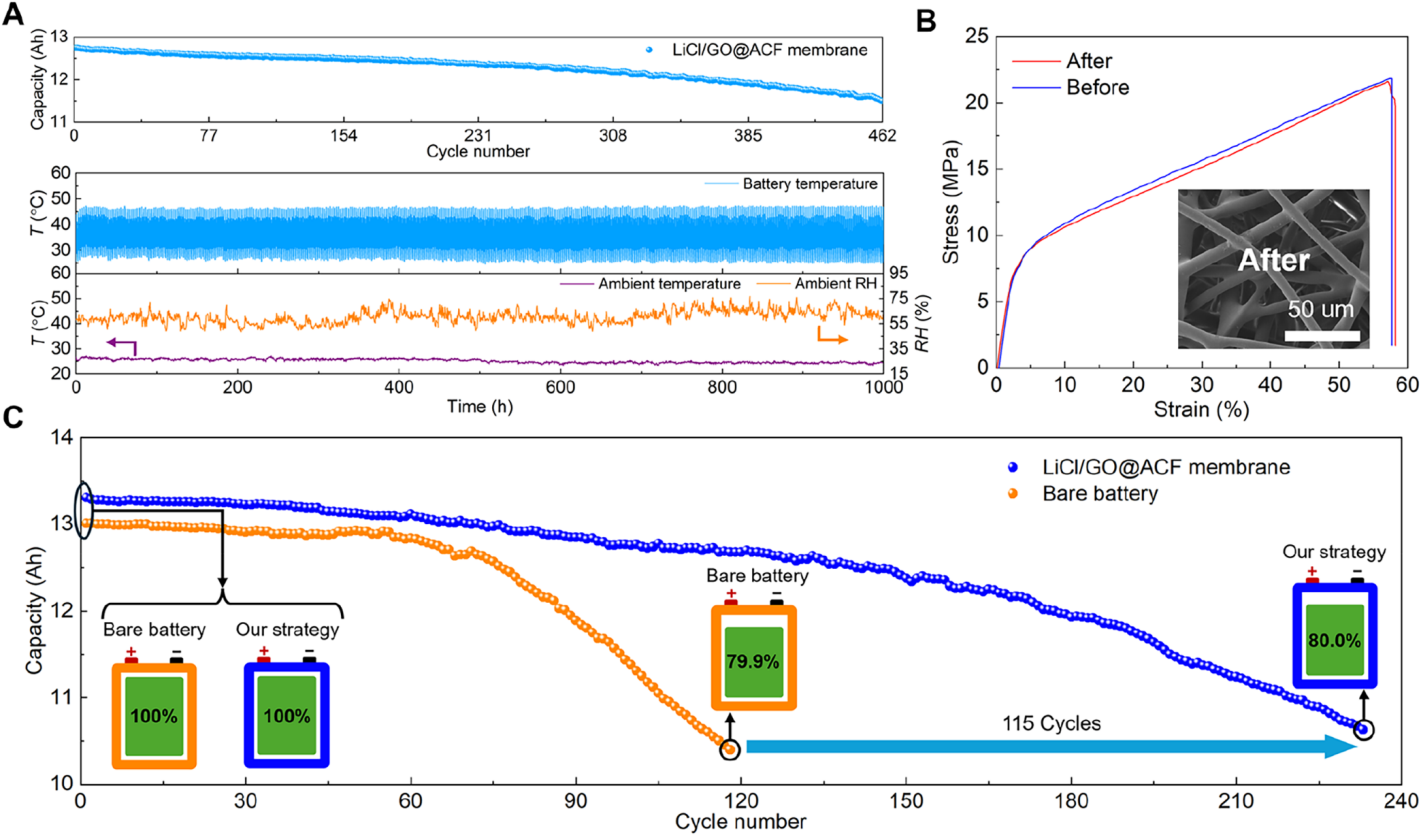
Cyclic performance demonstration on the 3.7 V/12 Ah LIB. (A) Capacity and temperature of the battery with LiCl/GO@ACF membrane at a long-term cyclic discharging-charging rate of 3C. (B) Comparison of the mechanical strength of the PTFE membrane. The inset shows the SEM of the PTFE membrane after cycling testing (more details in Figure S20). (C) Battery capacity decay with and without the LiCl/GO@ACF membrane at a cyclic discharging-charging rate of 4C.

We further evaluated the effect of the cooling membrane on the EoL point of LIBs at a high cyclic discharging-charging rate (RH = 65% and *T*_amb_ = 25 °C). Considering safety concerns, the testing was immediately interrupted once the battery temperature exceeded 75 °C. As shown in Figure 6C, the battery with the cooling membrane reaches its EoL point after 233 cycles, while the bare battery lasts only 118 cycles. Our cooling membrane significantly extends the LIB lifetime, contributing to a 2-fold improvement while providing an additional capacity of 1445.9 Ah. The remarkable performance improvement in the LIB demonstrates that our proposed cooling strategy is a highly promising candidate for enabling high-efficiency passive battery thermal management.

## Conclusions

In summary, we have proposed a high-efficiency, fire-resistant, and cost-effective passive thermal management for LIBs based on water desorption from a hygroscopic cooling membrane. Through morphological and compositional characterization, we have demonstrated that the LiCl/GO@ACF membrane possesses high water uptake capacity, high thermal conductivity, and excellent fire resistance. Based on the developed proof-of-concept experiments, we confirmed that the cooling performance of our proposed strategy significantly surpassed existing passive cooling strategies. Benefiting from the high evaporation enthalpy of water, a superior performance of the LiCl/GO@ACF membrane is expected in thermal management than traditional PCMs. Long-term cycling experiments (3C) confirmed that the LiCl/GO@ACF membrane still maintained desirable thermal management performance after 1000 h of continuous operation. At a high cyclic discharging-charging rate of 4C, our proposed cooling membrane extended LIB lifetime from 118 cycles to 233 cycles, achieving an additional total capacity of 1445.9 Ah, without corrosion or leakage. Furthermore, we developed an experimentally validated numerical model for optimization guidance. Our proposed strategy presents efficient cooling performance, excellent fire resistance, and low cost, offering a promising pathway for passive thermal management.

## Experimental Section

### Materials

Lithium chloride anhydrous (AR, 99.0%) was obtained from Dieckmann (HK) Chemical Industry Co., Ltd. Graphene oxide (GO) dispersion (5 mg/mL) was obtained from Hangzhou Gaoxi Technology Co., Ltd. PTFE membrane was obtained from HANGZHOU COBETTER FILTRATION EQUIPMENT Co., Ltd. Active carbon fiber (ACF) felt was purchased from Kunshan Longshengbao Electronic Materials Co., Ltd. 3.7 V/12 Ah LIBs were purchased from Phylion Battery Co., Ltd. PCM (Paraffin wax, 50–52 °C, 8002–74–2, Sinopharm Chemical Reagent Co., Ltd.). Acrylamide (AM, 99%), *N*,*N*′-methylenebis­(acrylamide) (MBA, 99%), ammonium persulfate (APS, 99%), and *N*,*N*,*N*′,*N*′-tetramethylethylendiamin (TEMED, 99%) were obtained from Dieckmann (HK) Chemical Industry Co., Ltd.

### Synthesis of the LiCl/GO@ACF

The desired amount of LiCl and GO dispersion was added into deionized water via magnetic stirring for 12 h to form a 25 mL homogeneous solution with 25% LiCl and 0.5–5.0% GO. To ensure the repeatability and accuracy of the experimental results, three identical samples were measured in each group. Specifically, the original ACF (90 mm × 90 mm × 2 mm) was immersed in the prepared LiCl/GO solution for 3, 6, 9, 12, and 15 h, respectively. Next, the obtained LiCl/GO@ACF samples were dried in a vacuum drying oven for 12 h at 80 °C. Then, the samples were placed on the electronic balance (METLER TOLEDO, ME503*T*/00) to obtain the mass of the dried samples. As shown in Table S7, we observed that shorter durations, such as 3 or 6 h, resulted in incomplete impregnation (i.e., a lower mass). Extending the immersion time beyond 12 h did not result in significant improvements in the mass, suggesting that 12 h was sufficient for achieving saturation. After that, the original ACF (46 mm × 46 mm) with different thicknesses was immersed in the prepared homogeneous LiCl/GO solution for 12 h to form LiCl/GO@ACF composite sorbent. To measure the stability of the LiCl/GO@ACF, the high-low temperature cycling aging tests were conducted. Specifically, prior to aging tests, the water uptake performance of the sample (46 mm × 46 mm × 2 mm) was measured 3 times under controlled conditions (10 h, 25 °C, 60% RH) to ensure reproducibility. The sample was then subjected to 100 high-low temperature cycles between −20 and 80 °C, with each temperature plateau maintained for 30 min. After aging, the water uptake performance of the sample was again measured 3 times under the same conditions.

### Synthesis of the LiCl@PAM

The PAM hydrogel was synthesized using a one-pot approach. Specifically, 6 g of acrylamide (AM) monomer was added to 30 mL of deionized water and stirred with a magnetic stirrer for 10 min until the AM monomer was fully dissolved. Subsequently, 60 mg of *N*,*N*′-methylenebis­(acrylamide) (MBA) as a cross-linker and 90 mg of ammonium persulfate (APS) as an initiator were added under continuous stirring. Finally, 30 μL of *N*,*N*,*N*′,*N*′-tetramethylethylendiamin (TEMED) was added as a cross-linking accelerator, and the resulting solution was allowed to cool to room temperature to form the PAM hydrogel. The obtained PAM hydrogel was washed with deionized water five times to remove homopolymers and unreacted monomers. To load LiCl, the sample was immersed in a 25 wt % LiCl solution for 24 h under ambient conditions (RH 60%, 25 °C), and the LiCl@PAM hydrogel was then placed in a prefabricated heat sink. After that, the hydrogel-based heat sink was frozen at −50 °C and vacuum-dried for 24 h. Before the comparison experiments, the dried LiCl@PAM hydrogel was placed in an environmental chamber (RH 60%, 25 °C) for 1 week to fully recover its water content (Figure S21).

### Materials Characterization

The thermal conductivity of the LiCl/GO@ACF with a thickness of 2 mm was measured using an isothermal conductive calorimeter (Hot Disk TPS 2500S). The obtained LiCl/GO@ACF was observed by scanning electron microscope (Thermo Scientific Apreo 2C) equipped with EDS (Oxford ULTIM Max 65). XRD spectra of the samples were measured by Rigaku Ultima IV. The contact angles were measured by a surface contact angle meter (JY-82C). XPS was measured by Thermo Scientific K-α. The long-term aging test was measured by ShockEvent T/60/V2. The desorption performance of the LiCl/GO@ACF was measured by thermogravimetric analysis (Rigaku TG-DTA 8122) and differential scanning calorimetry (DSC Q2000). The mechanical property of the PTFE membrane was measured by a universal mechanical test machine (INSTRON 3343) at a stretching rate of 0.5 mm min^–1^. The limiting oxygen index of the sample was tested by a limiting oxygen index tester (VOUCH 5801A) (see detailed information in Table S2). The combustion performance parameters of the sample were tested by a cone calorimeter (VOUCH 6810) at a power of 35 kW m^–2^.

## Supplementary Material







## Data Availability

The data that support the findings of this study are available from the corresponding author upon reasonable request.

## Notes

SEM, XPS, mechanical performance test, and cone calorimetric test of LiCl/GO@ACF and ACF; contact angle measurement; detailed experimental setup, numerical simulation, and cost analysis (PDF)
Flame-retardant test of the LiCl@ACF captured by a digital camera (MP4)
Flame-retardant test of the GO@ACF captured by a digital camera (MP4)
