# Supplementary material for: Mammal-Skin-Inspired Adaptive Nanocomposites Cooling Membrane for Passive Battery Thermal Management
Source: ACS Nano. 2025 Sep 1;19(36):32788–99. doi: 10.1021/acsnano.5c11130 (PMC12755196; doi:10.1021/acsnano.5c11130)
Supplement: Supplementary file 1 [file nn5c11130_si_001.pdf]

Supporting Information

Mammal-Skin-Inspired Adaptive Nanocomposites Cooling Membrane for Passive Battery Thermal Management

*Zengguang Sui, Jiaxiang Ma, Wei Wu**

School of Energy and Environment, City University of Hong Kong, Hong Kong 999077, China

Supporting Figures

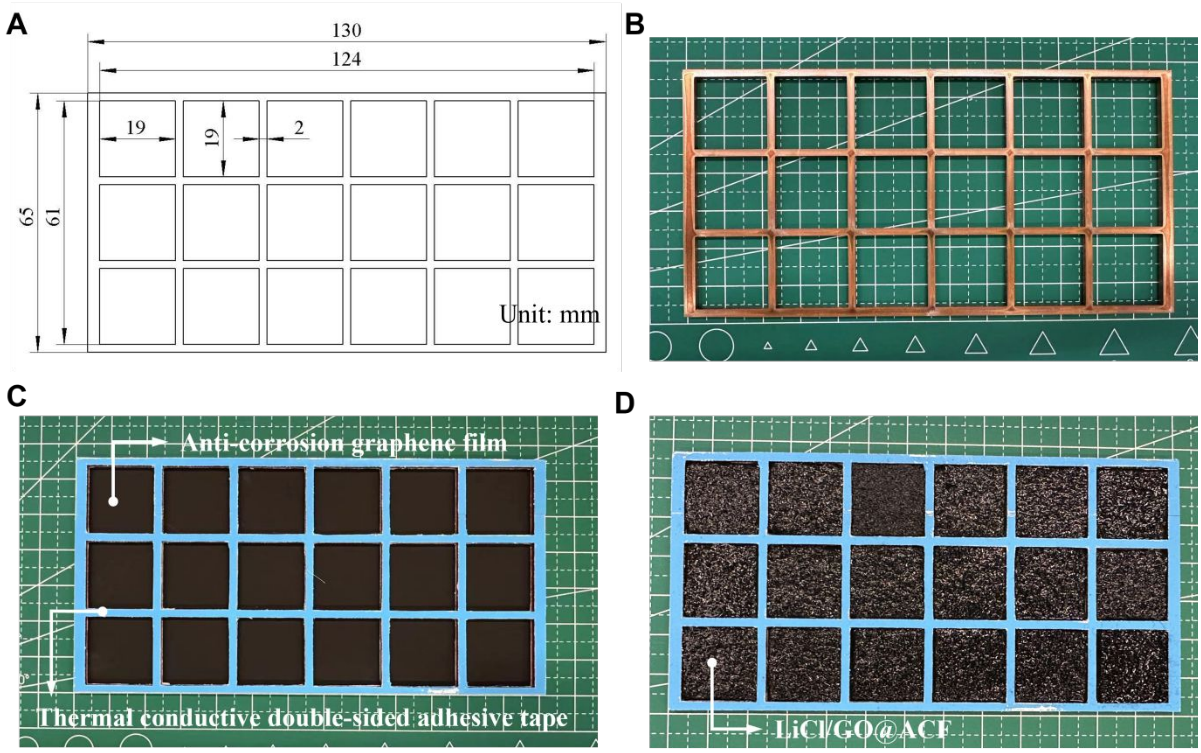

Figure S1. Photographs of the proposed cooling strategy. (A) Design parameters of the copper frame. (B) Photograph of the copper frame fabricated using laser cutting. (C) Photograph of the copper frame with anti-corrosion graphene film and thermal conductive tape. (D) LiCl/GO@ACF loaded the copper frame.

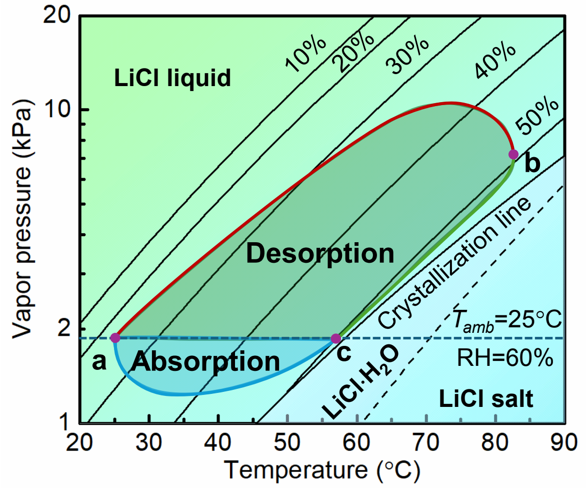

Figure S2. Desorption-absorption cycle in the pressure-temperature-concentration lines of the LiCl solution. a-b is the desorption process for dissipating the heat generated from LIBs; b-c represents that the cooling membrane undergoes a brief desorption process as p_s is still higher than p_v. c-a represents that the cooling membrane absorbs moisture from the surrounding atmosphere to autonomously recover cooling capacity.

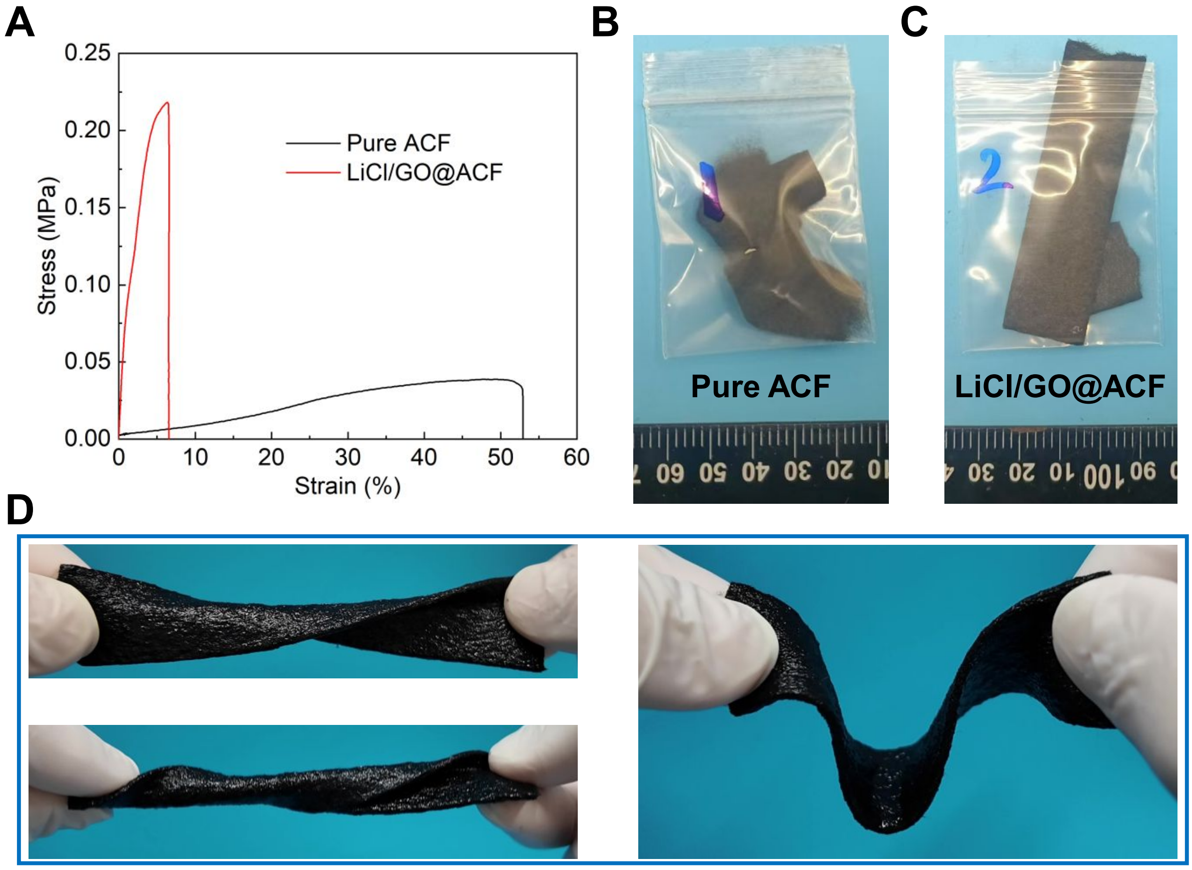

Figure S3. Mechanical performance testing of LiCl/GO@ACF. (A) Comparison of the mechanical strength of the pure ACF and dried LiCl/GO@ACF. (B) Pure ACF after mechanical performance testing. (C) Dried LiCl/GO@ACF after mechanical performance testing. (D) Photographs showing the shape adaptability of the LiCl/GO@ACF after moisture absorption (12 h).

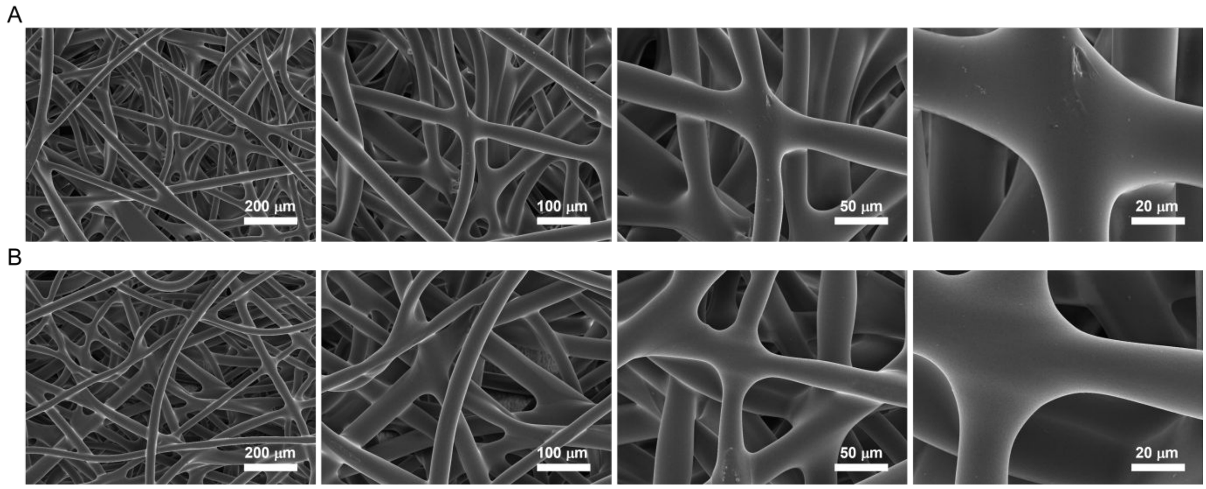

Figure S4. SEM images of PTFE membranes with a pore size of 1 μm (A) and 0.45 μm (B).

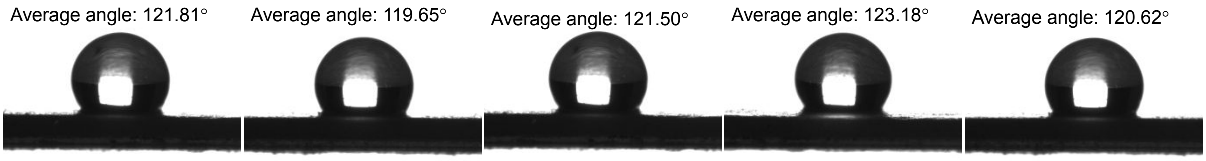

Figure S5. Contact angle measurement for 25 wt% LiCl solution on PTFE membrane, and the average contact angle is 121.35±1.32°.

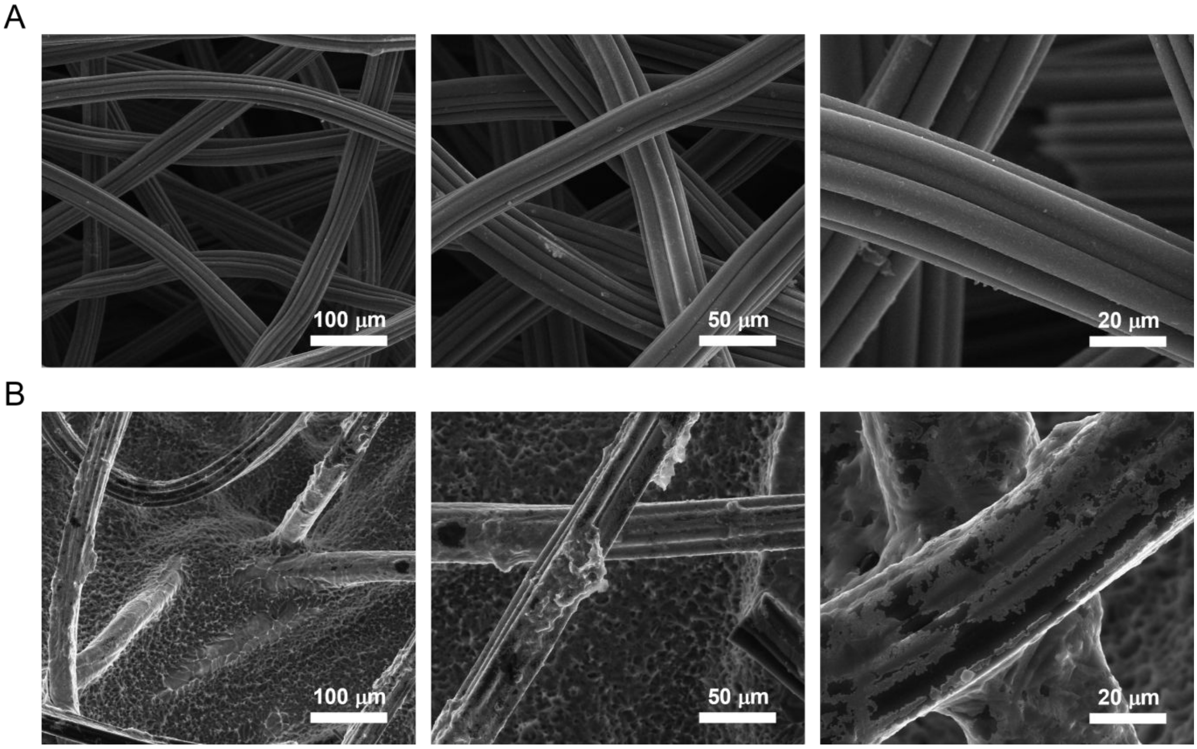

Figure S6. SEM images of the original ACF matrix (A) and LiCl/GO@ACF (B).

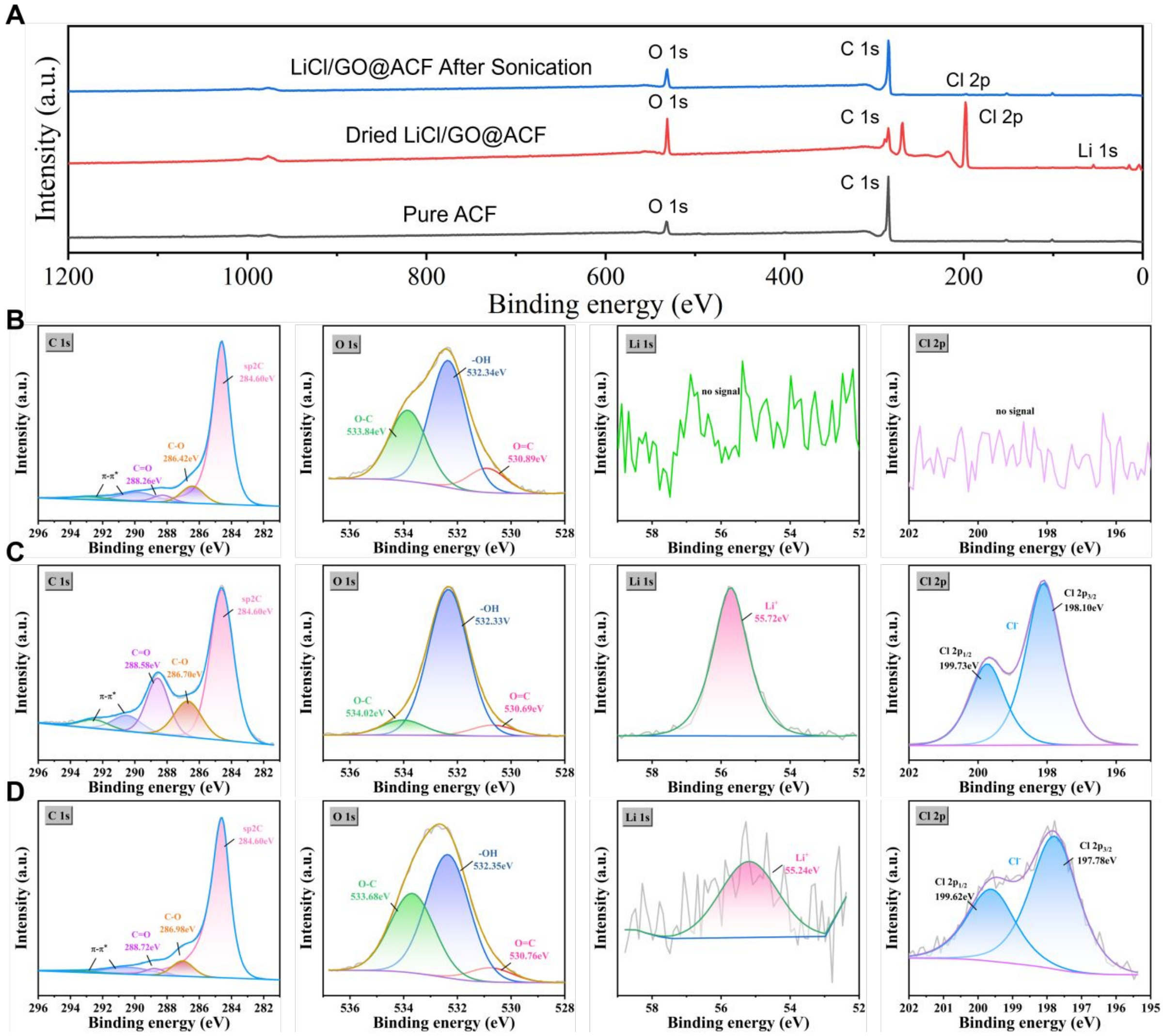

Figure S7. X-ray photoelectron spectra (XPS) for the pure ACF, dried LiCl/GO@ACF, and LiCl/GO@ACF after ultrasonication (0.5 h). (A) XPS for the pure ACF, dried LiCl/GO@ACF, and LiCl/GO@ACF after ultrasonication. (B) High-resolution XPS for the pure ACF. (C) High-resolution XPS for the dried LiCl/GO@ACF. (D) High-resolution XPS for the LiCl/GO@ACF after ultrasonication.

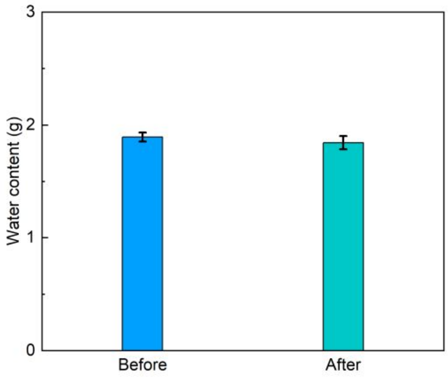

Figure S8. The water uptake capacities of the samples before and after the high-low temperature cycling aging test. Error bars represent the standard deviation (n=3).

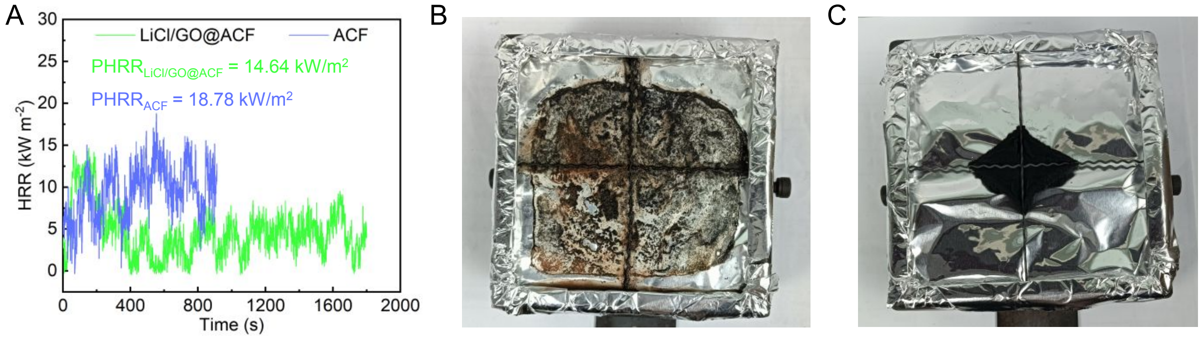

Figure S9. Cone calorimetric test results of LiCl/GO@ACF and ACF. (A) Heat release rate (HRR) of LiCl/GO@ACF. (B) Photograph of LiCl/GO@ACF after the cone calorimetry test. (C) Photograph of ACF after the cone calorimetry test.

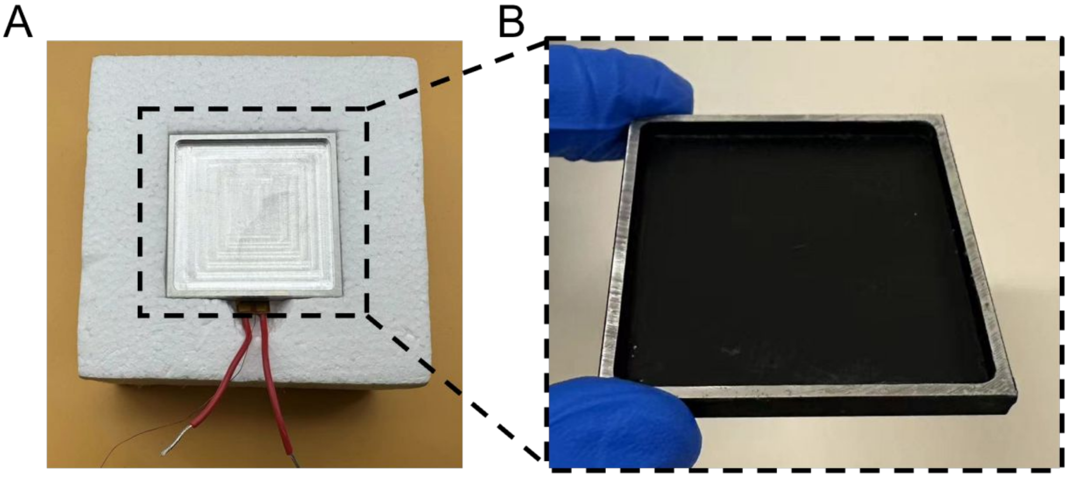

Figure S10. Photographs of the device under test to emulate the heat generated by LIBs. (A) Aluminum heat sink with the heater placed on the insulation foam to reduce the heat loss. (B) Aluminum heat sink with anti-corrosion graphene coating to prevent solution corrosion.

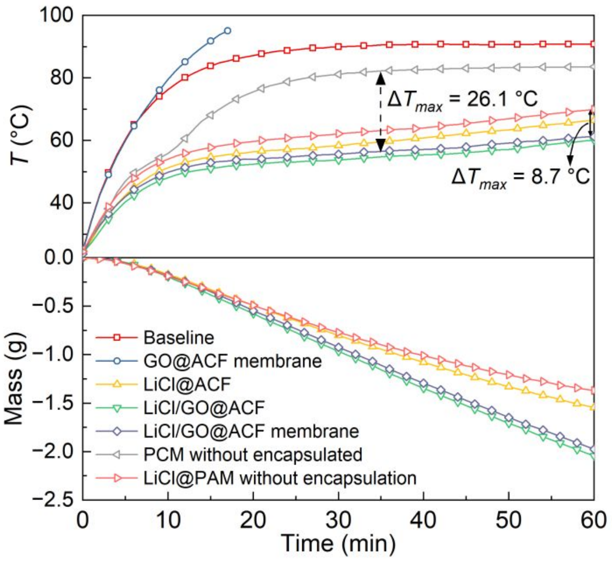

Figure S11. The temperature and mass evolutions of different cooling strategies at a heat flux of 2.0 kW m^{-2}. The baseline is shown in Figure S10.

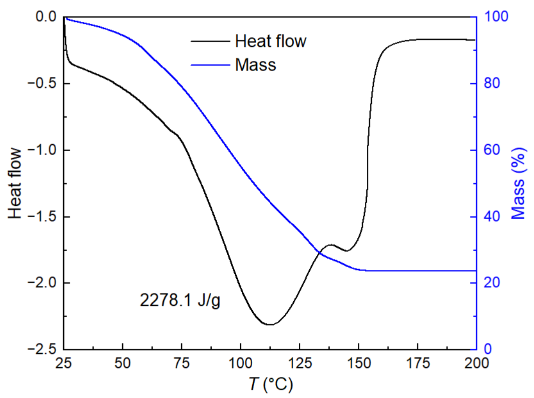

Figure S12. Differential scanning calorimetry and thermogravimetric analysis of the LiCl/GO@ACF.

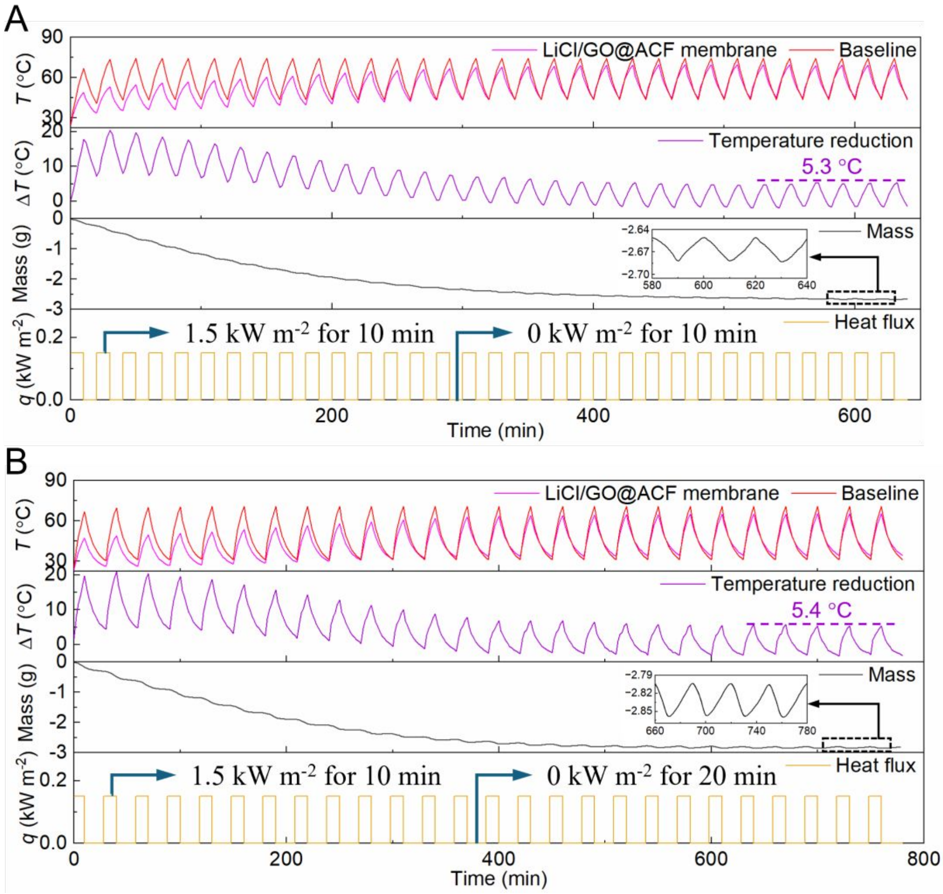

Figure S13. Cooling performance of the proposed strategy under periodic workloads at RH = 50% and T_{amb} = 25 °C. (A) Switching between 1.5 kW m^{-2} for 10 min and 0 kW m^{-2} for 10 min. (B) Switching between 1.5 kW m^{-2} for 10 min and 0 kW m^{-2} for 20 min.

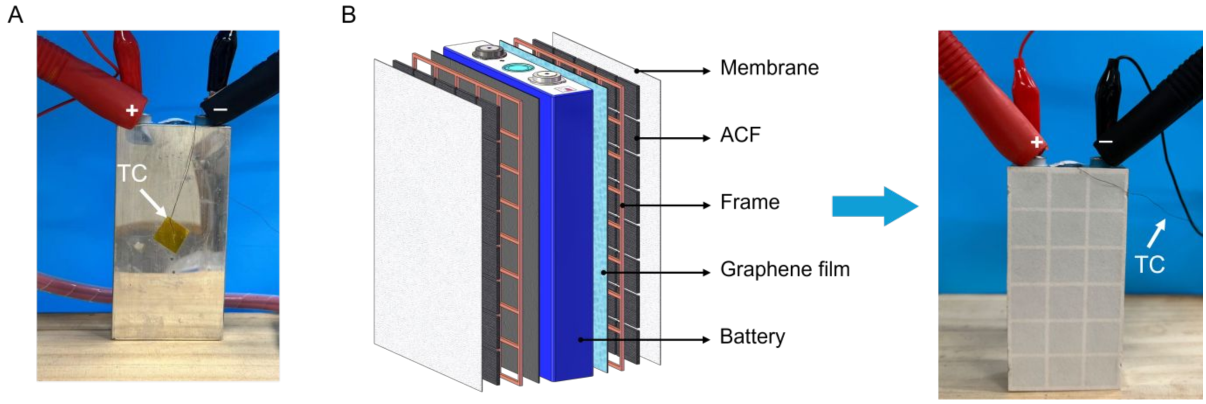

Figure S14. Photographs of the 3.7 V/12 Ah LIBs with and without our proposed cooling strategy. (A) 3.7 V/12 Ah LIB without the cooling strategy as the baseline. (B) 3.7 V/12 Ah LIB with the cooling strategy.

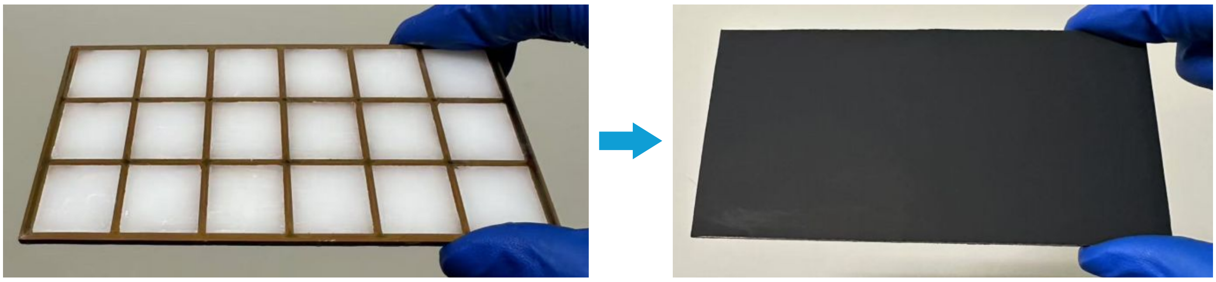

Figure S15. Photographs of the PCM-based cooling strategy encapsulated by graphene film.

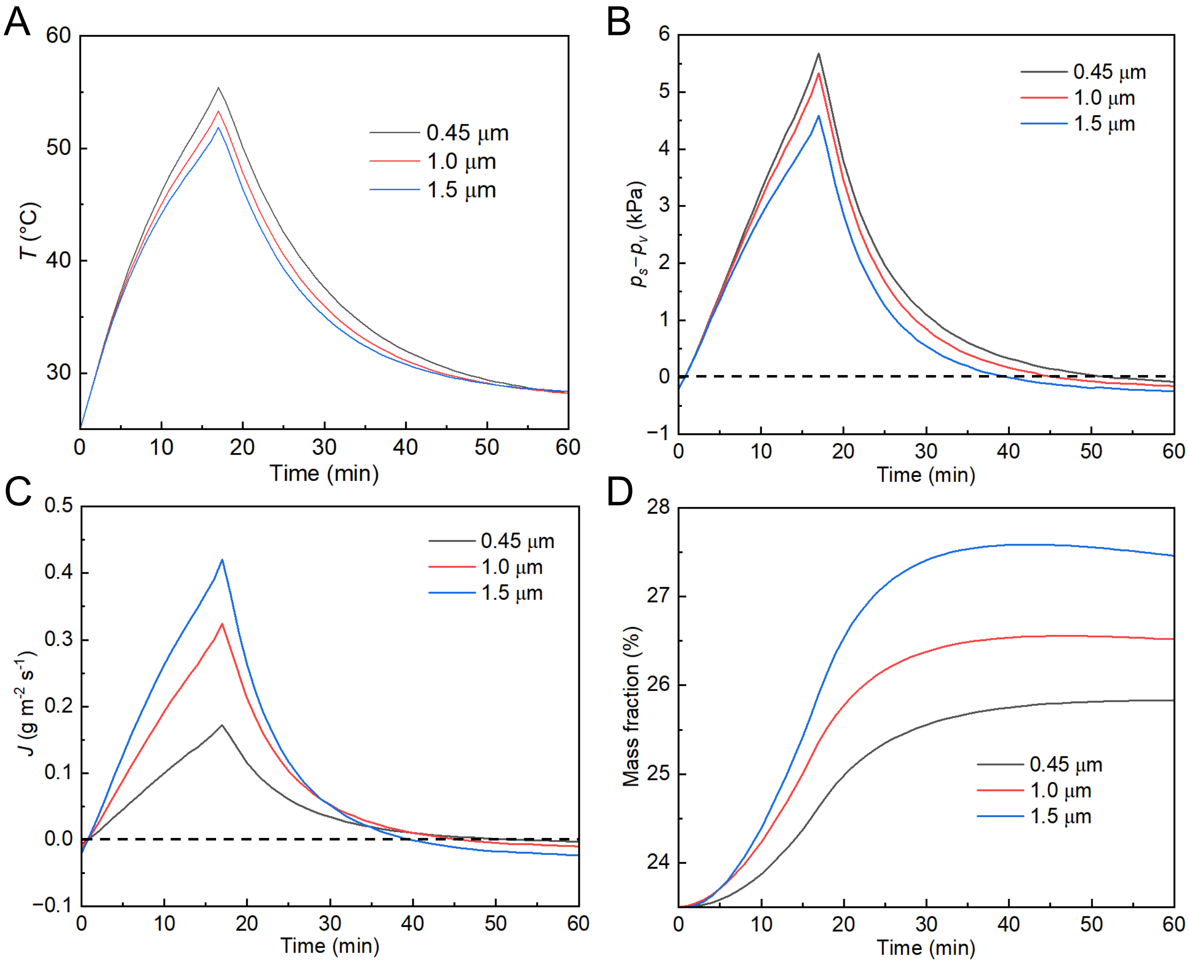

Figure S16. Simulated results with different membrane pore diameters at the discharging rate of 4C. (A) Battery temperature evolutions with different membrane pore diameters. (B) Membrane pressure potential ($p_s - p_v$) evolutions with different membrane pore diameters. (C) Membrane mass flux evolutions with different membrane pore diameters. (D) LiCl mass fraction evolutions with different membrane pore diameters.

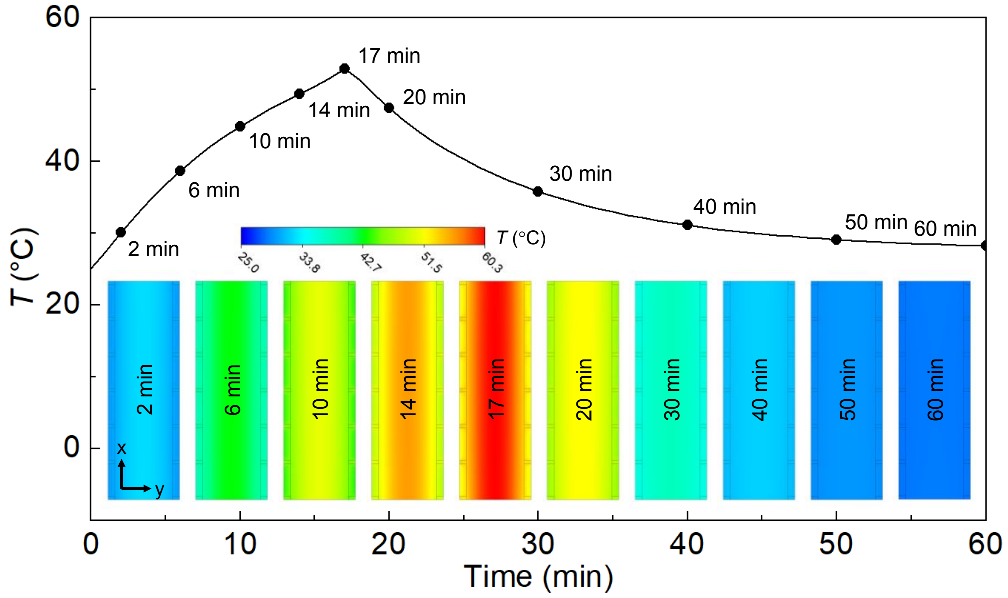

Figure S17. Temperature evolution and contour over time at the discharging rate of 4C. The scale factor is 0.5 in the x direction to show the contours more clearly.

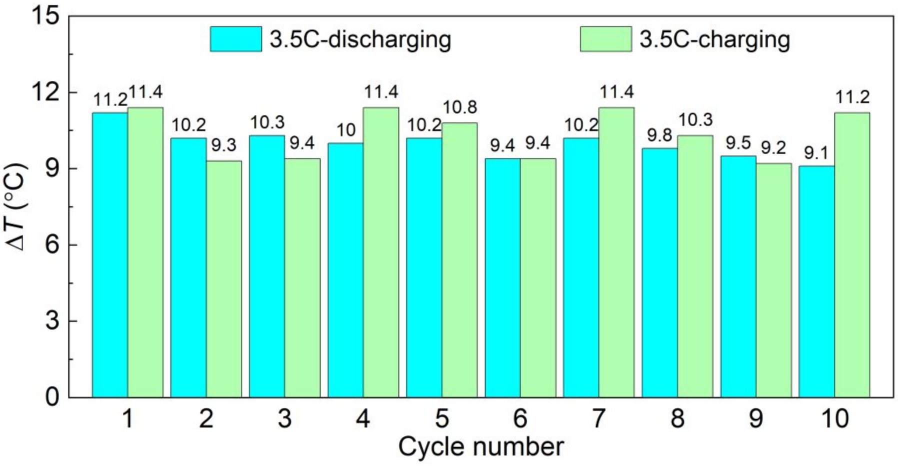

Figure S18. Peak temperature difference between the LIBs with and without the LiCl/GO@ACF membrane.

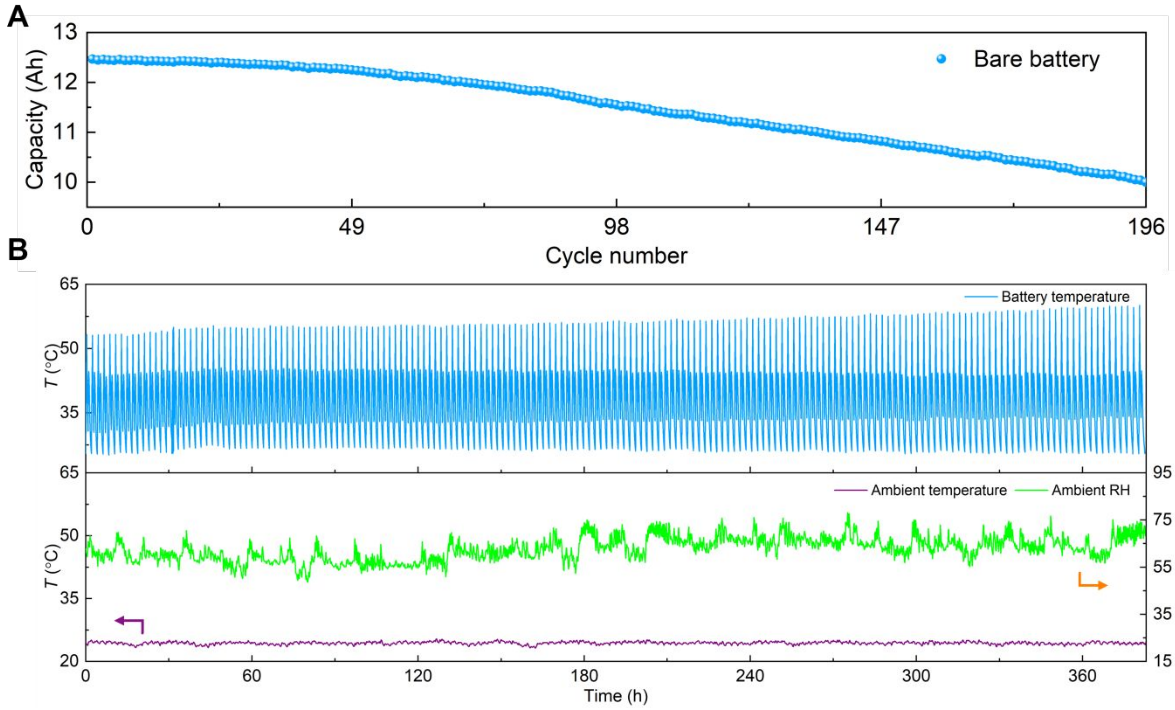

Figure S19. Capacity and temperature of the bare battery at a cyclic discharging-charging rate of 3C.

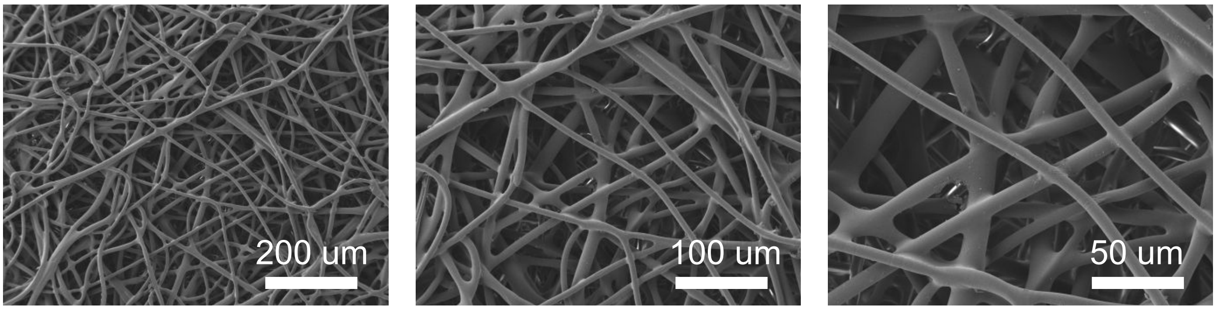

Figure S20. SEM images of PTFE membranes with a pore size of 1 μm after the cycling experiment at the discharging-charging rate of 3C.

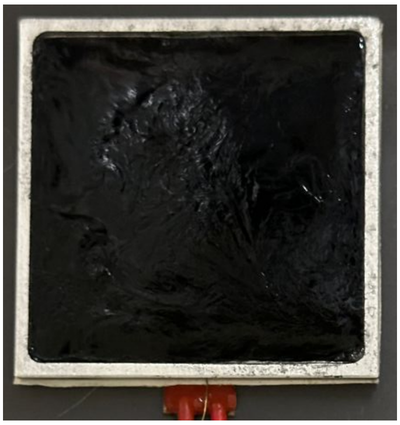

Figure S21. Photographs of the LiCl@PAM hydrogel.

Supporting Tables

Table S1. PTFE membrane parameters.

**Parameter**	**Value**
Thickness	0.3 mm
Pore diameter	1.0 μm
Porosity	80%
Thermal conductivity	0.22 W/m·k

Table S2. Experiment of limiting oxygen index.

Item	Parameter			
Test instrument	VOUCH 5801A			
Test environment	23 °C and RH = 50%			
Test standard	ISO4589			
Ambient gas	Oxygen, Nitrogen			
Sample size	80 mm × 20 mm × 2 mm			
Oxygen concentration (volume fraction)	30%	50%	70%	90%
Burning time (s)	0	0	0	0
Conclusion	Sample unburned			

Table S3. Combustion behavior parameters of LiCl/GO@ACF.

Sample	PHRR (kW m^{-2})	TTI (s)	TTF (s)	THR (MJ m^{-2})
LiCl/GO@ACF	14.64	/	/	8.31
Pure ACF	18.78	/	/	10.65

Note: PHRR is the peak heat release rate; TTI is the time to ignition; TTF is the time to flame; THR is the total heat release.

Table S4. Comparison of the LiCl/GO@ACF membrane with passive cooling strategies.

Object	Material	Operating condition		Cooling effect		H_{de}	C_E
		T_{amb} (°C)	RH (%)	ΔT_{max} (°C)	q_{de} W m^{-2}	(J g^{-1})	(USD kJ^{-1})
Electronic[1]	MOF-101(Cr)	25	60	8.6	312.5^b	1950	63.47
Electronic[2]	LiBr solution	25	60	13.0	409.1^b	2370	6.68×10^{-3}
Electronic[3]	LiCl@ACF	25	80	20.1	602.0^a	2400	4.17×10^{-3}
Electronic[4]	Li-PAAm	25	70	15.2	570.0^a	N/A	N/A
Electronic[5]	eMIL@PAN	30	60	7.7	436.0^b	1706	52.76
Electronic[6]	Hydrogel	22	60	13.0	400 W m^{-2}K^{-1}	2300	9.57×10^{-3}
Battery[7]	MOF-101(Cr)@CF	10	80	8.3	200.0^b	2541	48.71
Battery[8]	TG hydrogel	26	80	20.0	218.0^b	N/A	N/A
Battery[9]	DFCNT/HS	25	65	17.4	32.9 W m^{-2}K^{-1}	N/A	N/A
PV[10]	PAM-CB-LiCl	25	60	9.9	288.2^a	2450	0.03
PV[11]	PAN-CNT-$CaCl_2$	N/A	N/A	15.0	331.2^a	N/A	N/A
PV[12]	PAm/alginate-$CaCl_2$	30	75	7.5	140.0^b	N/A	1.17×10^{-3}
PV[4]	Li-PAAm	30	40	17.0	144.0^b	N/A	N/A
PV[13]	13X/H_2O/NH_4NO_3	N/A	N/A	15.1	403.0^a	1815	N/A
PV[14]	Hydrogel@CF	N/A	N/A	20.1	713.0^a	2450	N/A
PV[15]	Li-PAM	25	13	23.0	185.0^a	2450	1.17×10^{-3}
Building[16]	LiCl/SHEG	40	45	N/A	630.0^a	2450	N/A
This work	LiCl/GO@ACF	25	60	34.3	802.5^a	2278.1	7.77×10^{-3}
This work	PCM (Paraffin)	25	60	20.4	N/A	200	0.05

Note: "a" is the average cooling power, and "b" is the maximum cooling power.

Table S5. Nominal specification of the 3.7 V/12 Ah LIB.

Parameter	Specification
Battery dimension	$h_b \times l_b \times w_b$: 130 mm × 65 mm × 17 mm
Battery weight	300 g
Nominal capacity	12 Ah
Internal resistance	~3 mΩ
Nominal voltage	3.7 V
Charge cut-off voltage	4.2 V
Discharge cut-off voltage	2.75 V
Cut-off temperature	75 °C

Table S6. Cyclic discharging-charging test procedures.

Step	Mode	Parameter
Discharging	Constant current discharging	36 A
	Cut-off voltage	2.75 V
Rest	Rest	30 min
Charging	Constant voltage charging	4.2 V
	Cut-off current	0.5 A
Rest	Rest	30 min

Table S7. Immersion time testing of LiCl/GO@ACF.

Sample	3 h	6 h	9 h	12 h	15 h
#1	3.137	3.504	4.037	3.790	4.122
#2	3.315	3.692	3.751	4.101	3.967
#3	3.235	3.028	3.983	3.852	3.757
Average	3.229	3.408	3.924	3.914	3.949

Note S1. Experimental setup

1.1 Reaction equilibrium equation of $LiCl \cdot H_2O$

The chemical reaction equilibrium equation of $LiCl \cdot H_2O$ dehydration in Figure S2 can be calculated as Equation (S1)[17]:

$$\ln\frac{p_s}{p_0} = -\frac{8668.18}{(T+273.15)} - 4\ln(T+273.15) + 44.589 \tag{S1}$$

where p_0 = 101325 Pa.

1.2 Water vapor absorption experiments

To study the influence of GO content on the water uptake capacity of the LiCl/GO@ACF composite sorbent, 1 ml LiCl/GO homogenous solution with different GO content of 0-5% was added into the original ACF (46 mm × 46 mm × 2 mm), respectively. The obtained LiCl/GO@ACF samples were dried in a vacuum drying oven for 12 h at 80 °C. Then, the samples were placed on the electronic balance (METLER TOLEDO, ME503T/00) with a high resolution of 0.001 g in the environment-controlled chamber (RH = 60% and T_{amb} = 25 °C) for 20 h to record the mass change, respectively.

To measure the dynamic water uptake capacities of the LiCl/GO@ACF membrane (encapsulated with PTFE membrane) with different RH conditions, the original ACF (46 mm × 46 mm × 2 mm) was immersed in the prepared LiCl/GO solution for 12 h. The obtained LiCl/GO@ACF was placed on the aluminum heat sink (Figure S10) encapsulated with PTFE membrane to obtain a LiCl/GO@ACF membrane. Then, the LiCl/GO@ACF membrane was dried in a vacuum drying oven for 12 h at 80 °C. After that, the LiCl/GO@ACF membrane was placed on the electronic balance in the environment-controlled chamber to measure the mass change with different RH conditions. After the experiments, we measured the mass of the dried LiCl/GO@ACF, which was 1.118 g.

1.3 Proof-of-concept experiments

The desorption process and cooling performance of the proposed strategy were measured based

on a homemade test apparatus. In the proof-of-concept experiments, we used a (50 mm × 50 mm) polyimide resistive (PI) heater to emulate the heat generated by LIBs, and its heat flux can be adjusted by a direct-current power supplier (IT6831A). The prepared LiCl/GO@ACF membrane was placed into a CNC aluminum heat sink (Figure S10) and encapsulated using the PTFE membrane to measure its cooling performance. To prevent solution corrosion, the aluminum heat sink was coated using an anti-corrosion graphene. The heat sink was placed in an insulation foam (100 mm × 100 mm × 50 mm) with a thermal conductivity of 0.03 W m^{-1} K^{-1} to minimize heat loss. A super-fine K-type thermocouple (Omega) was mounted at the center of the heater to record the temperature evolution, and the calibrated TCs were connected to a data logger (Yakogawa, GM10-1C0). The device under test was placed on the electronic balance to measure the mass change, and all experiments were conducted in the environment-controlled chamber.

1.4 Demonstration experiments in real LIBs

To demonstrate the cooling performance of the proposed strategy in real applications, the commercial 3.7 V/12 Ah nickel-cobalt-manganese LIBs were prepared. A TC was placed on the surface of the tested LIB. The LIB was connected to a battery charging/discharging tester (YPSDZ-0550). For charging mode, the tester was set to a constant voltage charging of 4.2 V and a cut-off current of 0.5 A. The discharging mode was set to a cut-off voltage of 2.75 V. Considering the safety issues, the testing would be stopped when the surface temperature of the LIBs exceeded 75 °C. Before the experiments, the LIBs with and without the cooling strategy were first fully charged and naturally cooled to 25 °C in the environment-controlled chamber, then they were measured at different modes. During the experiments, an infrared camera (Fluke, Ti60+) was used to visualize the surface temperature of the LIBs. The flame retardancy test was conducted using a lighter (LEHMAN), which was recorded by a movie camera (Osmo Pocket 3).

1.5 Uncertainty analysis

Measuring sensors and instruments used will influence the experimental results. Uncertainty analysis is thus conducted to calculate the accuracy of the experimental results. We conducted

the uncertainty analysis according to the method reported by Moffat[18]. Assuming that R can be calculated from directly measured parameters v_1, v_2, v_3, ···, v_n as a function:

$$R = f\left(v_1, v_2, v_3, \cdots, v_n\right) \tag{S2}$$

The relative uncertainties can be evaluated as follows:

$$u\left(R\right) = \sqrt{\left(\frac{\partial f}{\partial v_1} u\left(v_1\right)\right)^2 + \left(\frac{\partial f}{\partial v_2} u\left(v_2\right)\right)^2 + \cdots + \left(\frac{\partial f}{\partial v_n} u\left(v_n\right)\right)^2} \tag{S3}$$

Note S2. Cost analysis

To evaluate the increased cost when applying the LiCl/GO@ACF membrane in the real LIB (3.7 V/12 Ah), we conduct the cost analysis based on the used raw materials (i.e., LiCl, ACF, GO, and PTFE membrane). Notably, it is not easy to accurately assess the cost of a material that has not yet been commercialized, but the raw materials used to fabricate the proposed cooling membrane are common and inexpensive materials. Therefore, we use the selling prices of these materials on a global online procurement platform to assess the raw material costs of the cooling membrane. The following table lists the price of each material, and all prices are provided by verified suppliers on the platform.

**Material**	**Unit Price**	**Material source**
LiCl	13.06 USD/kg	Dieckmann (HK) Chemical Industry Co., Ltd
ACF	31.78 USD/kg	Kunshan Longshengbao Electronic Materials Co., Ltd
GO	27.51 USD/kg	Hangzhou Gaoxi Technology Co., Ltd
PTFE membrane	2.80 USD/m^2	HANGZHOU COBETTER FILTRATION EQUIPMENT Co., Ltd
Total	11.53 USD/m^2	

As illustrated in the table, the raw material cost of the cooling membrane with a thickness of 2 mm is about 11.53 USD m^{-2}, which is 5.31% of the LIB price (3.58 USD).

Note S3. Numerical simulation

3.1 Governing equations

We simulated the heat and mass transfer process of the tested battery equipped with the proposed cooling strategy using CFD tool FLUENT. As shown in the following figure, the computational domains include the solid domains (battery and copper frame) and the liquid domain (LiCl solution), and the ACF was simulated using a porous media model. The detailed parameters of the copper frame and battery cell can be found in Figure S1 and Table S5, respectively.

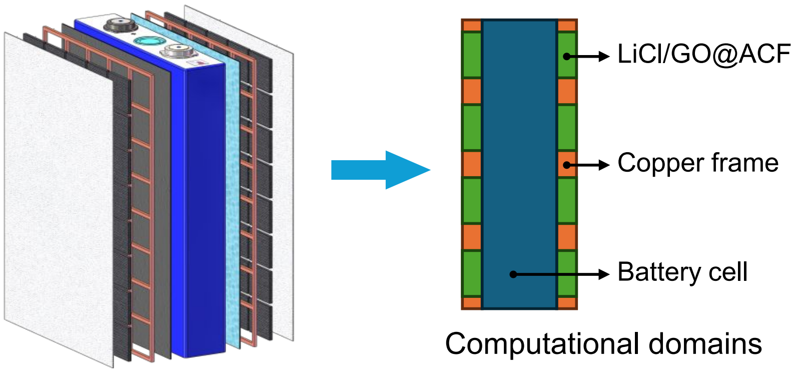

The water vapor through the PTFE membrane was considered to be the heat and mass sources, which can be added to governing equations using user-defined functions. To simplify the simulation, the relative velocity between the species is negligible, and the volume of salt solution is assumed constant during desorption and absorption. Notably, we mainly discuss the heat and mass transfer through the porous membrane, the detailed simulation can be found in our previous work[2]. The overall governing equations include the mass, energy, momentum, and species transport equations as follows:

Continuity equation $$\nabla \cdot \left(\rho \vec{v} \right) = S_m \tag{S4}$$

Momentum equation $$\nabla \cdot \left(\rho \vec{v}\vec{v} \right) = -\nabla p + \nabla \cdot \left(\bar{\bar{\tau}} \right) + \rho \vec{g} + \vec{F} \tag{S5}$$

Energy equation $$\nabla \cdot \left(\vec{v}\left(\rho E + p \right) \right) = \nabla \cdot \left(k_{eff} \nabla T - \sum_i h_i \vec{J}_i + (\bar{\bar{\tau}}_{eff} \cdot \vec{v}) \right) + S_h \tag{S6}$$

Species equation $$\nabla \cdot \left(\rho \vec{v} Y_i \right) = \nabla \cdot \left(\rho D_{vapor-solution} \nabla Y_i \right) + S_i \tag{S7}$$

where $\vec{v}$ is the velocity vector; ρ is the density; S_m is the additional mass source term and indicates the mass flux of water vapor through the membrane; p is the pressure; $\vec{F}$ is the external body forces; $\rho\vec{g}$ is the gravitational force; $\bar{\bar{\tau}}$ is the stress tensor; h_i is the enthalpy of the species i; k_{eff} is the effective conductivity; T is the temperature; $\vec{J}_i$ is the diffusion flux of species i; S_h is the heat source term; S_i is the specie source term; $D_{vapor\text{-}solution}$ is the mass diffusion coefficient.

3.2 Mass transfer model

The water vapor mass flux through the porous PTFE membrane can be calculated as[19]:

Absorption process $$J = k_m\left(p_v - p_s\right) \tag{S8}$$

Desorption process $$J = k_m\left(p_s - p_v\right) \tag{S9}$$

where J is the mass transfer flux; p_s and p_v are the equilibrium water vapor partial pressure of the solution and the water vapor pressure, respectively; p_s is determined by the solution temperature and concentration and can be calculated by the method from Ref[20]:

$$p_s = p_{H_2O}(1-\left(1+\left(\frac{x}{0.362}\right)^{-4.75}\right)^{-0.4} - 0.03e^{-\frac{(x-0.1)^2}{0.005}})f \tag{S10}$$

where x is the mass fraction; f is defined as:

$$f = A + B\theta \tag{S11}$$

$$A = 2-\left(1+\left(\frac{x}{0.28}\right)^{4.3}\right)^{0.6} \tag{S12}$$

$$B = \left(1+\left(\frac{x}{0.21}\right)^{5.1}\right)^{0.49} - 1 \tag{S13}$$

$$\theta = \frac{T}{T_{c,H_2O}} \tag{S14}$$

In Equation (S10), p_{H2O} is the vapor pressure of water, which can be defined as:

$$\ln\left(\frac{p_{H_2O}}{p_{c,H_2O}}\right) = \frac{A_0\tau + A_1\tau^{1.5} + A_2\tau^3 + A_3\tau^{3.5} + A_4\tau^4 + A_5\tau^{7.5}}{1-\tau} \tag{S15}$$

$$\tau = 1 - \frac{T}{T_{c,H_2O}} \tag{S16}$$

where $T_{c,H2O}$ is the critical temperature of water in Kelvin (647.096 K).

According to the Dusty-Gas model, the mass transfer coefficient through the porous membrane k_m can be simplified as[19]:

$$k_m = \frac{2\varepsilon}{3\lambda \cdot \delta_m} d_p \sqrt{\frac{2M_{H_2o}}{\pi R T_m}} \tag{S17}$$

$$\lambda = \frac{(2\text{-}\varepsilon)^2}{\varepsilon} \tag{S18}$$

where ε is the membrane porosity; δ_m is the membrane thickness; T_m is the average temperature of the membrane; ε is the membrane porosity; M_{H_2O} and R are the water molecular weight and the universal gas constant, respectively; λ is the pore tortuosity factor; The detailed membrane parameters can be found in Table S1.

3.3 Heat transfer model

In this cooling strategy, the heat transfer of water vapor desorbed from the solution involves the water vapor enthalpy (h_v), the solution enthalpy (h_s)[21], and the dilution heat (h_d)[20]. Therefore, the heat source S_h is given by:

$$S_h = J(h_v - h_s + \Delta h_d) \tag{S19}$$

$$h_d = h_{d,0}\left[1 + \left(\frac{w}{H_1}\right)^{H_2}\right]^{H_3} \tag{S20}$$

$$w = \frac{x}{H_4 - x} \tag{S21}$$

$$h_{d,0} = H_5 + H_6\theta \tag{S22}$$

The parameters H_i for Equation (S20)-(S22) are given in the following table.

**Parameter**	H_1	H_2	H_3	H_4	H_5	H_6
**Value**	0.845	−1.965	−2.265	0.6	169.105	457.850

For the battery cell, S_h is the heat generated from the battery cell, which can be expressed as:

$$S_h = \frac{Q_{gen}}{V_c} \tag{S23}$$

where V_c is the volume of the battery cell; Q_{gen} is the heat generation of the battery composed of the reversible heat and the irreversible heat, which can be defined as:

$$Q_{gen} = I^2 R - I T_{cell} \frac{dU_{oc}}{dT_{cell}} \tag{S24}$$

where I, R, T_{cell}, and U_{oc} denote the current, internal resistance, battery cell temperature, and open-circuit voltage, respectively. The details can be found in our previous work[22].

3.4 Numerical treatment

We used structural quadrilateral grids to mesh the computational domains, and the inflation layers were used near the PTFE membrane to accurately capture heat and mass transfer characteristics. In our previous studies, the desorption and absorption processes of lithium bromide solution have been simulated successfully[19,22,23]. The heat and mass transfer processes of LiCl solution and LiBr solution are very similar. Therefore, the desorption and absorption processes of the LiCl/GO@ACF membrane could be developed based on the numerical models of LiBr solution and the thermophysical properties of LiCl solution[20].

For the tested battery, the heat generation in the battery is assumed to be uniform, which mainly includes the irreversible heat and reversible heat. The modeling process has been discussed in detail in our previous work, where we conducted a multi-objective optimization to maximize the cooling performance of a liquid cooling-based battery thermal management system[22]. All simulations were conducted on a Windows-based server with two 2.30 GHz Intel(R) Xeon(R) Gold 5218 CPUs and 128 GB of RAM.

References

1. Wang, C., Hua, L., Yan, H., Li, B., Tu, Y., and Wang, R. (2020). A Thermal Management Strategy for Electronic Devices Based on Moisture Sorption-Desorption Processes. Joule *4*, 435-447.
2. Sui, Z., Sui, Y., Ding, Z., Lin, H., Li, F., Yang, R., and Wu, W. (2023). Membrane-encapsulated, moisture-desorptive passive cooling for high-performance, ultra-low-cost, and long-duration electronics thermal management. Device *1,* 100121.
3. Liu, H., Yu, J., Wang, C., Zeng, Z., Poredoš, P., and Wang, R. (2023). Passive thermal management of electronic devices using sorption-based evaporative cooling. Device *1*, 100122.
4. Pu, S., Fu, J., Liao, Y., Ge, L., Zhou, Y., Zhang, S., Zhao, S., Liu, X., Hu, X., Liu, K., and Chen, J. (2020). Promoting Energy Efficiency via a Self-Adaptive Evaporative Cooling Hydrogel. Advanced Materials *32*, e1907307.
5. Liu, X., Li, P., Chen, J., Jiang, P., Mai, Y.-W., and Huang, X. (2022). Hierarchically porous composite fabrics with ultrahigh metal organic framework loading for zero-energy-consumption heat dissipation. Science Bulletin *67*, 1991-2000.
6. Zeng, J., Zhang, X., Chung, K.M., Feng, T., Zhang, H., Prasher, R.S., and Chen, R. (2023). Moisture thermal battery with autonomous water harvesting for passive electronics cooling. Cell Reports Physical Science *4*, 101250.
7. Xu, J., Chao, J., Li, T., Yan, T., Wu, S., Wu, M., Zhao, B., and Wang, R. (2020). Near-Zero-Energy Smart Battery Thermal Management Enabled by Sorption Energy Harvesting from Air. ACS Central Science *6*, 1542-1554.
8. Pu, S., Liao, Y., Chen, K., Fu, J., Zhang, S., Ge, L., Conta, G., Bouzarif, S., Cheng, T., Hu, X., et al. (2020). Thermogalvanic Hydrogel for Synchronous Evaporative Cooling and Low-Grade Heat Energy Harvesting. Nano Letters. *20*, 3791-3797.
9. Zhang, L., Yu, W., Wang, J., Gao, D., Chen, Y., Dai, W., Wang, P., Li, G., Meng, C., Liu, C., and Guo, S. (2023). Carbon Nanotube/Hygroscopic Salt Nanocomposites with Dual-Functionality of Effective Cooling and Fire Resistance for Safe and Ultrahigh-Rate Operation of Practical Lithium-Ion Batteries. Advanced Functional Materials *33*, 2213846.
10. Liu, Y., Liu, Z., Wang, Z., Sun, W., and Kong, F. (2024). Photovoltaic passive cooling via water vapor sorption-evaporation by hydrogel. Applied Thermal Engineering *240*, 122185.
11. Li, R., Shi, Y., Wu, M., Hong, S., and Wang, P. (2020). Photovoltaic panel cooling by atmospheric water sorption–evaporation cycle. Nature Sustainability *3*, 636-643.
12. Li, Z., Ma, T., Ji, F., Shan, H., Dai, Y., and Wang, R. (2023). A Hygroscopic Composite Backplate Enabling Passive Cooling of Photovoltaic Panels. ACS Energy Letters *8*, 1921-1928.
13. Kim, S., Park, J.H., Lee, J.W., Kim, Y., and Kang, Y.T. (2023). Self-recovering passive cooling utilizing endothermic reaction of NH4NO3/H2O driven by water sorption for photovoltaic cell. Nature Communications *14*, 2374.
14. Wang, W.-W., Chen, J.-W., Zhang, C.-Y., Yang, H.-F., Ji, X.-W., Zhang, H.-L., Zhao, F.-Y., and Cai, Y. (2024). Green thermal management of photovoltaic panels by the absorbent hydrogel evaporative (AHE) cooling jointly with 3D porous copper foam (CF) structure. Energy *293*, 130467.
15. Zou, W., Ji, M., Han, C., Tian, E., and Mo, J. (2024). Enhancing the internal thermal conductivity of hydrogel for efficient passive heat dissipation: Experimental study of a surface simulating a cooled photovoltaic panel. Energy Conversion and Management *306*, 118328.
16. Lin, W., Yao, X., Kumar, N.M., Lo, W.K., Chopra, S.S., Hau, N.Y., and Wang, S. (2024).

Camel-Fur-Inspired Graphite-Based Hygroscopic Membrane for Passive Air Cooling with Ultrahigh Cooling Power. Advanced Energy Materials *14*, 2303470.

17. Xu, J., Li, T., Chao, J., Wu, S., Yan, T., Li, W., Cao, B., and Wang, R. (2020). Efficient Solar-Driven Water Harvesting from Arid Air with Metal-Organic Frameworks Modified by Hygroscopic Salt. Angewandte Chemie International Edition *59*, 5202-5210.
18. Moffat, R.J. (1988). Describing the uncertainties in experimental results. Experimental thermal and fluid science *1*, 3-17.
19. Sui, Z., Wu, W., You, T., Zheng, Z., and Leung, M. (2021). Performance investigation and enhancement of membrane-contactor microchannel absorber towards compact absorption cooling. International Journal of Heat and Mass Transfer *169*, 120978.
20. Conde, M.R. (2004). Properties of aqueous solutions of lithium and calcium chlorides: formulations for use in air conditioning equipment design. International Journal of Thermal Sciences *43*, 367-382.
21. Pátek, J., and Klomfar, J. (2008). Thermodynamic properties of the LiCl–H2O system at vapor–liquid equilibrium from 273K to 400K. International Journal of Refrigeration *31*, 287-303.
22. Sui, Z., Lin, H., Sun, Q., Dong, K., and Wu, W. (2024). Multi-objective optimization of efficient liquid cooling-based battery thermal management system using hybrid manifold channels. Applied Energy *371*, 123766.
23. Sui, Z., Zhai, C., and Wu, W. (2022). Parametric and comparative study on enhanced microchannel membrane-based absorber structures for compact absorption refrigeration. Renewable Energy *187*, 109-122.
